# Gait-phase specific transverse-plane momenta generation during pre-planned and late-cued 90 degree turns while walking

**DOI:** 10.1038/s41598-023-33667-1

**Published:** 2023-04-26

**Authors:** Mitchell Tillman, Janine Molino, Antonia M. Zaferiou

**Affiliations:** 1 Department of Biomedical Engineering, Stevens Institute of Technology, Castle Point on the Hudson, Hoboken, NJ 07030 USA; 2 Department of Orthopaedics, Warren Alpert Medical School of Brown University/Rhode Island Hospital, Providence, RI USA; 3 Lifespan Biostatistics, Epidemiology, and Research Design Core, Rhode Island Hospital, Providence, RI USA

**Keywords:** Biomedical engineering, Motor control, Sensorimotor processing

## Abstract

Turning while walking is ubiquitous and requires linear and angular momenta generation to redirect the body’s trajectory and rotate towards the new direction of travel. This study examined strategies that healthy young adults used during each gait phase to generate transverse-plane momenta during pre-planned and late-cued 90° turns. During leftward turns, we expected that momenta would be generated most during the gait phases known to generate leftward linear and angular momenta during straight line gait. We found distinct roles of gait phases towards generating momenta during turns that partially supported our hypotheses. Supporting one hypothesis, the change in transverse-plane angular momentum and average moment were greater during double support with the left foot in front vs. other gait phases. Also, the change in leftward linear momentum and average leftward force were greater during right single support vs. other gait phases during straight-line gait and late-cued turns. However, during pre-planned turns, the average leftward force was not significantly greater during right single support vs. other gait phases. Overall, transverse-plane angular momentum generation during turns is similar to its generation during straight-line gait, suggesting that healthy young adults can leverage momenta control strategies used during straight-line gait during turns.

## Introduction

Up to half of all steps during walking are not part of straight-line gait and 90° turns often occur during daily walking tasks^1^. In everyday mobility, turns can occur in a pre-planned manner, when the person knows prior to the turn execution that they will need to complete the turn, or in a late-cued manner, when the cue to turn is provided by the perception of an environmental cue. To accomplish a turn, multiple mechanical objectives must be managed at the whole-body level: balancing, redirecting the body center of mass (COM) trajectory, and reorienting the body facing direction. These objectives are accomplished during walking turns via whole-body linear and angular momenta regulation that takes place across multiple gait phases. Turns can be accomplished with diversity in the number and placement of steps when performed by healthy adults and those with balance impairments^2–11^. For instance, we found that during pre-planned and late-cued 90° turns, healthy adults used from two to five steps and that their footfalls occurred in different locations relative to the intersection when using different turn strategies^2^.

**Figure 1:**
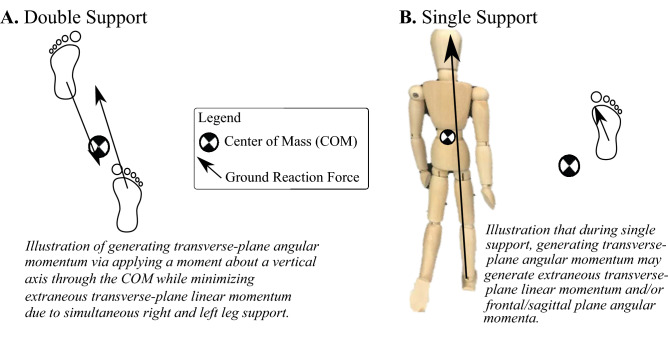
Illustration to explain the mechanical context to generate transverse-plane angular momentum during (**a**) double support, when both feet contact the ground vs. (**b**) single support, when only one foot is in contact with the ground**.** During double support, two legs can work together to avoid extraneous linear impulse generated by each of their ground reaction forces. In contrast, during single support, the linear momentum generated by the ground reaction force from the leg in contact with the ground cannot be concurrently neutralized by the other leg (because the other leg is not in contact with the ground), which can lead to extraneous linear momentum generation.

With this diversity of footfall patterns used during different types of turns (e.g., pre-planned and late-cued turns, etc.), it is our goal to better understand if there are underlying momenta control patterns that are used consistently within each gait phase, as in straight-line gait. Each gait phase offers specific mechanical contexts that can support or hinder achieving certain balance, linear, and angular mechanical objectives as the body alternates from single and double support in upright locomotion^12–15^. For example, applying a moment about a vertical axis to generate angular impulse during double support (both feet in contact with the ground) allows two legs to work together to avoid extraneous linear impulse (conceptual illustration in Fig. 1)^16,17^. The distinct mechanical contexts of single and double support gait phases may afford consistent momenta generation patterns across different walking tasks. If we uncover consistent roles of each gait phase towards generating required changes in momenta during different types of turns performed by healthy adults, we can inform locomotor diagnostic or rehabilitation practices. For instance, if healthy individuals usually generate requisite momenta within specific gait phase contexts during different types of turns, we can compare these “normative” gait-phase specific patterns to those used by populations who struggle to turn.

Prior research provides initial indications that motor control patterns during turning maneuvers may mimic those in straight-line gait. In recent computational research, Desmet, Cusumano, and Dingwell demonstrated that humans control foot placement during side-stepping (a maneuver used during turns) by modulating stepping regulation strategies used during straight-line gait, rather than switching to use a distinctly different control strategy^21^. In prior turning research, the COM trajectory has been observed to oscillate side-to-side across gait phases at certain turning speeds relative to an overall curved path. These side-to-side oscillations are similar to the sinusoidal path observed during straight-line gait^11,22^. Thus, there may be oscillatory momenta control patterns during turns that are shared with those used during straight line gait, despite notable differences in the overall COM trajectory and balance patterns^2,8,23^.

Linear momenta generation patterns within gait-phases are not yet known during walking turns. During straight-line gait, side-to-side linear momentum oscillates and allows shifting weight towards the side of the body taking the next step. During right single support, linear momentum redirects from rightwards to leftwards in preparation for the upcoming left heel strike^24,25^. During turns, linear momentum must be generated and sustained in the new direction of travel. Prior work has investigated changes in ground reaction forces and impulses during turns for the entirety of stance phase (when each foot is in contact with the ground) to accomplish changes in direction^22,23,26–28^. However, this prior work did not distinguish between single vs. double support phases of gait, despite the distinctly different base of support contexts provided by these sub-phases.

Similar to linear momenta, angular momenta generation patterns within gait-phases are not yet known during walking turns. During straight line gait, the transverse-plane whole-body angular momentum oscillates about zero. Transverse-plane angular momentum peaks early single support (near or just after toe-off), and crosses zero approximately when heel strike events occur, starting double support^25,29,30^. During turns, transverse-plane angular momentum is generated towards the desired facing direction^31,32^, but it is unclear how this is managed within each gait phase. Prior turning research in athletic maneuvers has revealed that generating angular impulse by redirecting ground reaction forces during double support facilitates mitigating extraneous linear impulse and avoiding conflicts between rotation and balance maintenance or COM translational goals^16,17,33,34^. For instance, during single support, if a large moment is applied from the ground reaction force, this force needs to be directed away from the COM, which may accelerate the COM in an undesired direction, or a direction away from the base of support (Fig. 1b). During cutting maneuvers and other athletic turns, athletes often need to generate linear and angular momenta during single support and do so in body configurations that are not statically stable (i.e., the COM is outside of the horizontal boundaries of the base of support)^35,36^. In contrast, during double support, the extraneous mediolateral or anteroposterior forces can concurrently “cancel” across limbs, preventing extraneous COM acceleration or angular momenta in the frontal and sagittal planes, while generating desired linear and angular momenta (Fig. 1a)^16,17,33^.

The purpose of this study was to examine strategies used by healthy young adults during each gait phase to accomplish the transverse-plane mechanical objectives of pre-planned and late-cued 90° turns, both of which occur as we walk through different environments. We hypothesized that within each task (1) straight-line gait, (2) pre-planned turns to the left, and (3) late-cued turns to the left: (a) during right single support phase, the greatest change in leftward linear momentum (∆p_x_) and average leftward force (F_x,avg_) are generated to redirect the COM trajectory towards the new direction of travel, and (b) during double support phases with the left foot in front of the body (left double support), the greatest change in transverse-plane angular momentum (∆Hz) and average transverse-plane moment (M_z,avg_) are generated to rotate the body to the desired facing direction.

## Results

### Linear mixed model omnibus results

Omnibus statistical test results are included in Table 1. These results indicated a significant effect of gait phase on ∆H_z_, M_z,avg_, and ∆p_x_, but not on F_x,avg_, which prompted post-hoc analysis within each task across gait phases. The lack of statistical significance for the Task* Gait phase interaction for F_x,avg_ suggests that the effect of gait phase on F_x,avg_ does not differ by task. However, we decided to present the results stratified by task for F_x,avg_ as well because 0.072 is close to 0.05 and indicates a potential effect, even if it is weak. Further discussion of the statistical interpretation of Task*Gait phase interaction effect is in Supplemental Document 2.

**Table 1:** Statistical results for the original linear mixed model (omnibus test). Significant values are in bold.

Fixed effect	Momentum variable							
	∆H_z_		M_z,avg_		∆p_x_		F_x,avg_	
	F	p	F	p	F	p	F	p
Task	F(2, 108) = 0.32	0.727	F(2, 108) = 0.64	0.528	F(2, 108) = 107.80	**< 0.0001**	F(2, 108) = 158.09	**< 0.0001**
Gait phase	F(3, 108) = 301.02	**< 0.0001**	F(3, 108) = 316.11	**< 0.0001**	F(3, 108) = 32.42	**< 0.0001**	F(3, 108) = 50.87	**< 0.0001**
Task * Gait phase	F(6, 108) = 6.43	**< 0.0001**	F(6, 108) = 5.62	**< 0.0001**	F(6, 108) = 12.96	**< 0.0001**	F(6, 108) = 2.00	0.072

### Global leftward (−X) change in linear momentum (∆p_x_) and average force (F_x,avg_)

**Figure 2:**
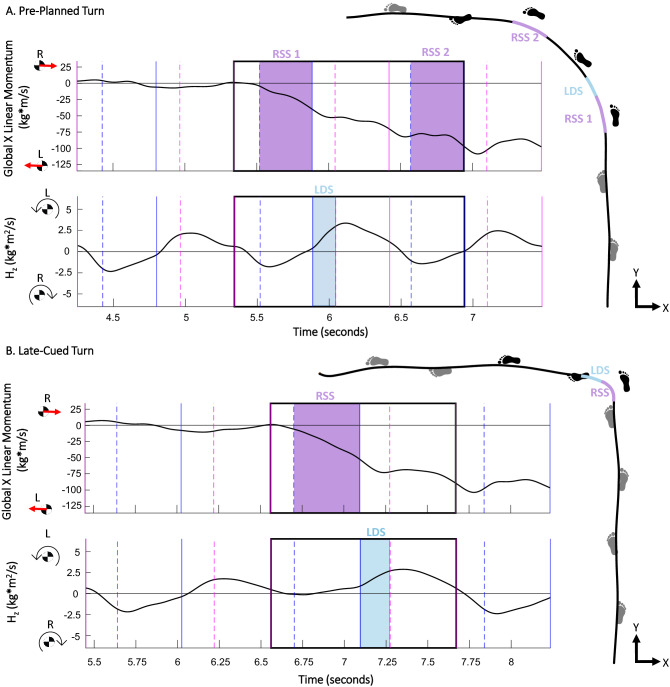
Example timeseries of the global X-axis linear momentum and transverse-plane angular momentum (H_z_) during (**a**) a pre-planned turn and (**b**) a late-cued turn. Vertical solid lines indicate heel-strike events and vertical dashed lines indicate toe-off events for the right foot (pink) and left foot (blue). The black-outline boxed region indicates the turn phase and color-filled boxes highlight right single support (RSS; purple) and left double support (LDS; blue) gait phases. These two trials provide examples of when the largest change in global leftward X-axis linear momentum occurred during RSS and the largest change in H_z_ occurred during LDS. Transverse-plane plots are included to provide context of the COM path and footfall locations.

**Figure 3:**
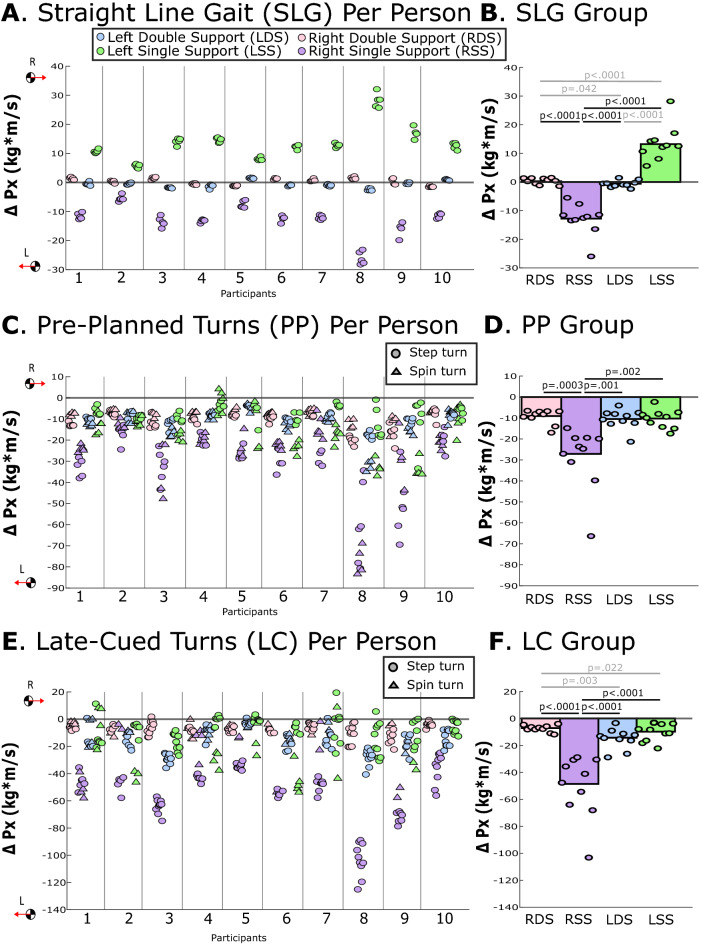
The change in global X-axis linear momentum (∆p_x_) for each gait phase during (**A**,**B**) straight-line gait, (**C**,**D**) pre-planned turns, and (**E**,**F**) late-cued turns. Negative p_x_ is leftward in the direction of the turn. The left panel (**A**,**C**,**E**) provides average ∆p_x_ for each gait phase, each trial, and each participant, with step turns indicated with circles and spin turns indicated with triangles. In the right panel (**B**,**D**,**F**), each circle indicates the average value for each participant across multiple trials and the bar represents the across-participant (group) averages. Horizontal lines with p-values indicate statistically significant differences between gait phases at the group level, those in gray are significant differences that were not related to the hypothesis.

**Figure 4:**
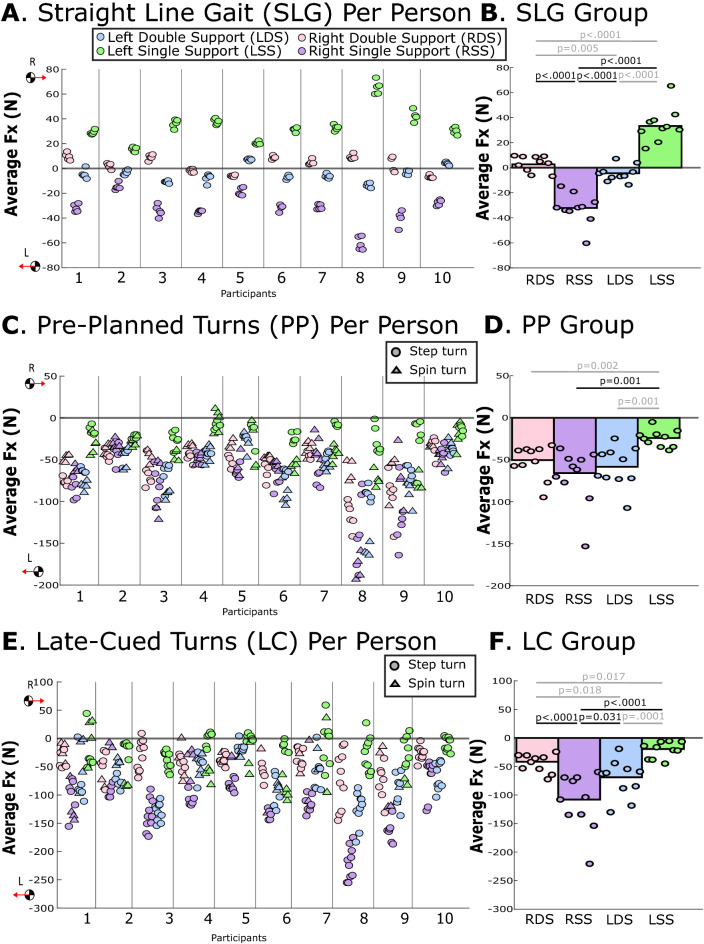
The average global X-axis force (F_x,avg_) for each gait phase during (**A**,**B**) straight-line gait, (**C**,**D**) pre-planned turns, and (**E**,**F**) late-cued turns. Negative F_x,avg_ is leftward in the direction of the turn. The left panel (**A**,**C**,**E**) provides average F_x,avg_ for each gait phase, each trial, and each participant, with step turns indicated with circles and spin turns indicated with triangles. In the right panel (**B**,**D**,**F**), each circle indicates the average value for each participant across multiple trials and the bar represents the across-participant (group) averages. Horizontal lines with p-values indicate statistically significant differences between gait phases at the group level, those in gray are significant differences that were not related to the hypothesis.

During straight-line gait and during late-cued turns, changes in leftward ∆p_x_ and leftward F_x__,avg_ were significantly greater during the right single support phase vs. any other phase (p-values ≤ 0.031; Table 2, Figs. 2, 3, 4). During pre-planned turns, leftward ∆p_x_ was significantly greater during right single support vs. any other phase (p-values ≤ 0.002; Table 2, Fig. 3). During pre-planned turns, leftward F_x,avg_ was significantly greater during right single support vs. left single support (p = 0.001), but not significantly different from left and right double support phases (p = 0.873, p = 0.483, respectively; Table 2**,** Fig. 4). Within-participant measures are displayed in Figs. 3 and 4 and within-participant statistical results are included in Supplemental Document 2.

**Table 2:** Group-level estimated marginal means for study linear outcomes from linear mixed models. Acronyms: Left Double Support (LDS) Left Single Support (LSS), Right Double Support (RDS), Right Single Support (RSS), change in global X-axis linear momentum (∆p_x_), and global X-axis average force (F_x,avg_), where negative X is leftward (the direction of the turn). Bolded p-values indicate statistically significant differences. Italic text indicates comparisons that were not related to the right single support phase that was pertinent to the hypothesis.

	Task	Estimated marginal mean (95% CI)				Post-hoc comparisons, Cohen’s d and p-values					
		LDS	LSS	RDS	RSS	LDS v. RSS	RSS v. LSS	RSS v. RDS	LSS v. RDS	LSS v. RSS	RDS v. RSS
∆p_x_ (kg m/s)	Straight-line gait	− 0.64 (− 1.34, 0.07)	13.59 (9.96, 17.22)	0.36 (− 0.30, 1.02)	− 12.95 (− 16.22, − 9.67)	− *3.27* *** < 0.0001***	− *0.85* ***0.042***	3.10 ** < 0.0001**	*3.05* *** < 0.0001***	4.60 ** < 0.0001**	3.35 ** < 0.0001**
	Pre-planned turns	− 11.22 (− 14.29, − 8.14)	− 12.41 (− 15.61, − 9.21)	− 9.84 (− 11.90, − 7.78)	− 29.53 (− 38.48, − 20.58)	*0.15* *0.923*	− *0.27* *0.923*	1.44 **0.001**	− *0.35* *0.550*	1.24 **0.002**	1.58 **0.0003**
	Late-cued turns	− 15.72 (− 20.37, − 11.07)	− 13.00 (− 16.95, − 9.05)	− 7.47 (− 8.96, − 5.97)	− 51.22 (− 64.68, − 37.76)	− *0.22* *0.379*	− *1.12* ***0.003***	1.89 ** < 0.0001**	− *0.50* ***0.022***	1.86 ** < 0.0001**	2.45 ** < 0.0001**
F_x,avg_ (N)	Straight-line gait	− 4.59 (− 8.37, − 0.82)	34.07 (25.97, 42.17)	3.18 (− 0.58, 6.93)	− 32.50 (− 39.87, − 25.12)	− *3.66* *** < 0.0001***	− *1.20* ***0.005***	2.81 ** < 0.0001**	*2.92* *** < 0.0001***	5.13 ** < 0.0001**	3.59 ** < 0.0001**
	Pre-planned turns	− 62.64 (− 78.46, − 46.82)	− 29.18 (− 36.59, − 21.77)	− 54.82 (− 66.78, − 42.86)	− 71.70 (− 92.16, − 51.24)	− *1.28* ***0.001***	− *0.28* *0.873*	0.26 0.873	*1.09* ***0.002***	1.35 **0.001**	0.51 0.483
	Late-cued turns	− 75.08 (− 95.85, − 54.31)	− 26.18 (− 34.81, − 17.56)	− 44.39 (− 53.86, − 34.92)	− 114.36 (− 143.26, − 85.46)	− *1.28* ***0.0001***	− *0.85* ***0.018***	0.80 **0.031**	*0.56* ***0.017***	1.91 ** < 0.0001**	1.57 ** < 0.0001**

### Transverse-plane angular momentum and moment

**Figure 5:**
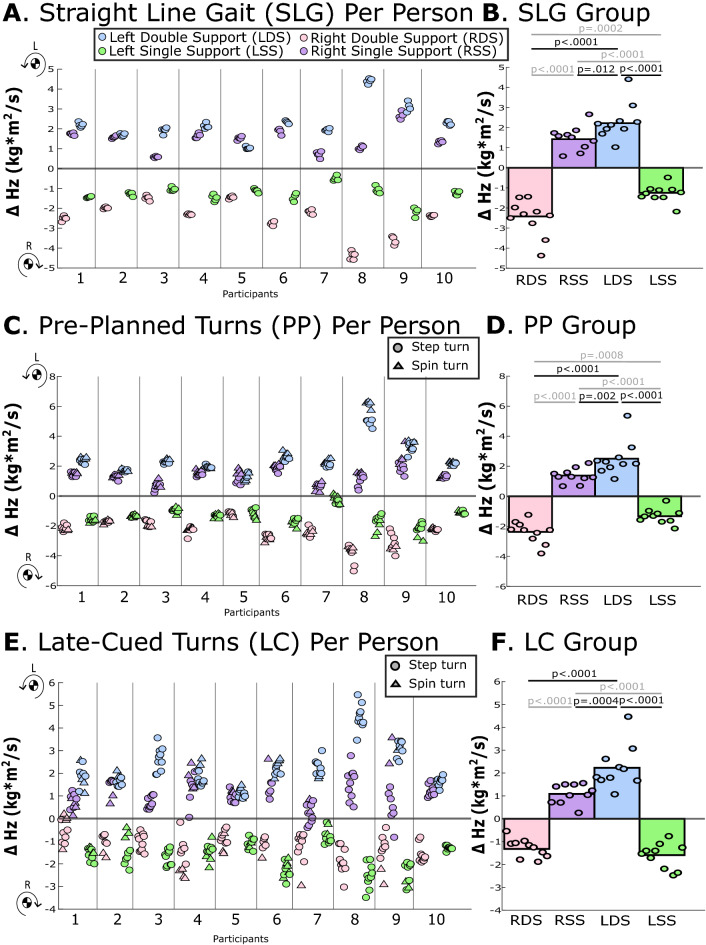
The average change in transverse-plane angular momentum (∆H_z_) for each gait phase during (**A**,**B**) straight-line gait, (**C**,**D**) pre-planned turns, and (**E**,**F**) late-cued turns. Positive H_z_ is leftward in the direction of the turn. The left panel (**A**,**C**,**E**) provides average ∆H_z_ for each gait phase, each trial, and each participant, with step turns indicated with circles and spin turns indicated with triangles. In the right panel (**B**,**D**,**F**), each circle indicates the average value for each participant across multiple trials and the bar represents the across-participant (group) averages. Horizontal lines with p-values indicate statistically significant differences between gait phases at the group level, those in gray are significant differences that were not related to the hypothesis.

**Figure 6:**
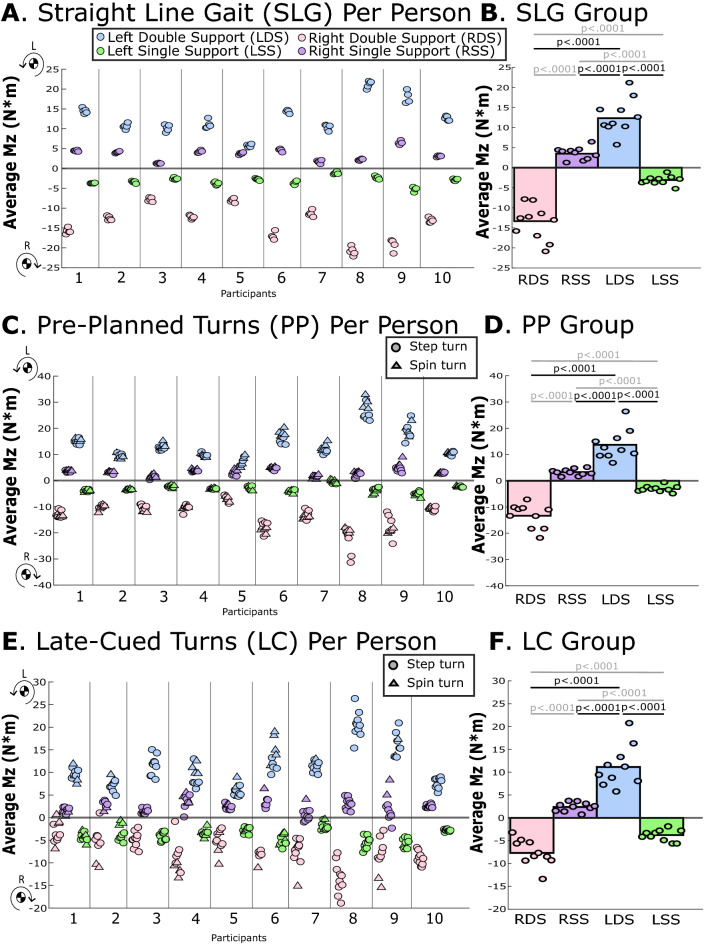
The average transverse-plane moment about a vertical axis through the COM (M_z,avg_) for each gait phase during (**A**,**B**) straight-line gait, (**C**,**D**) pre-planned turns, and (**E**,**F**) late-cued turns. Positive M_z,avg_ is leftward in the direction of the turn. The left panel (**A**,**C**,**E**) provides average M_z,avg_ for each gait phase, each trial, and each participant, with step turns indicated with circles and spin turns indicated with triangles. In the right panel (**B**,**D**,**F**), each circle indicates the average value for each participant across multiple trials and the bar represents the across-participant (group) averages. Horizontal lines with p-values indicate statistically significant differences between gait phases at the group level, those in gray are significant differences that were not related to the hypothesis.

During straight-line gait, pre-planned and late-cued turns, ∆H_z_ and M_z,avg_ were significantly greater during left double support (when the left leg was forward) vs. any other phase (p-values ≤ 0.012; Table 3**,** Figs. 2, 5, 6). Within-participant measures are displayed in Figs. 5 and 6 and within-participant statistical findings are included in Supplemental Document 2.

**Table 3:** Group-level estimated marginal means for study angular outcomes from linear mixed models. Acronyms: Left Double Support (LDS) Left Single Support (LSS), Right Double Support (RDS), Right Single Support (RSS), change in transverse-plane angular momentum (∆H_z_) and average transverse-plane moment (M_z_). Positive H_z_ and M_z_ rotate the body towards the left, which is the direction of the turn in pre-planned and late-cued turns. Italic text indicates comparisons that were not related to the left double support phase that was pertinent to the hypothesis. Bolded p-values indicate statistically significant differences.

	Task	Estimated marginal mean (95% CI)				Post-hoc comparisons Cohen’s d and p-values					
		LDS	LSS	RDS	RSS	LDS v. LSS	LDS v. RDS	LDS v. RSS	LSS v. RDS	LSS v. RSS	RDS v. RSS
∆H_z_ (kg m^2^/s)	Straight-line gait	2.30 (1.77, 2.84)	− 1.28 (− 1.53, − 1.02)	− 2.50 (− 3.04, − 1.96)	1.47 (1.11, 1.83)	5.18 ** < 0.0001**	5.45 ** < 0.0001**	1.12 **0.012**	*1.78* ***0.0002***	− *5.53* *** < 0.0001***	− *5.37* *** < 0.0001***
	Pre-planned turns	2.54 (1.82, 3.26)	− 1.35 (− 1.68, − 1.01)	− 2.34 (− 2.76, − 1.92)	1.33 (1.05, 1.61)	4.27 ** < 0.0001**	5.00 ** < 0.0001**	1.35 **0.002**	*1.48* ***0.0008***	− *4.83* *** < 0.0001***	− *5.58* *** < 0.0001***
	Late-cued turns	2.27 (1.72, 2.83)	− 1.66 (− 2.02, − 1.30)	− 1.25 (− 1.46, − 1.03)	1.09 (0.83, 1.35)	4.71 ** < 0.0001**	4.22 ** < 0.0001**	1.39 **0.0004**	− *0.62* *0.054*	− *4.02* *** < 0.0001***	− *3.43* *** < 0.0001***
M_z,avg_ (Nm)	Straight-line gait	12.85 (10.25, 15.44)	− 3.13 (− 3.77, − 2.49)	− 13.76 (− 16.36, − 11.17)	3.58 (2.65, 4.50)	5.12 ** < 0.0001**	6.24 ** < 0.0001**	2.89 ** < 0.0001**	*3.44* *** < 0.0001***	− *5.22* *** < 0.0001***	− *5.46* *** < 0.0001***
	Pre-planned turns	13.75 (10.17, 17.33)	− 3.14 (− 3.97, − 2.30)	− 12.84 (− 15.14, − 10.53)	3.06 (2.34, 3.78)	3.99 ** < 0.0001**	4.99 ** < 0.0001**	2.52 ** < 0.0001**	*2.77* *** < 0.0001***	− *4.61* *** < 0.0001***	− *4.53* *** < 0.0001***
	Late-cued turns	11.20 (8.68, 13.73)	− 3.95 (− 4.77, − 3.13)	− 7.42 (− 8.85, − 5.99)	2.49 (1.88, 3.10)	4.36 ** < 0.0001**	4.29 ** < 0.0001**	2.49 ** < 0.0001**	*1.15* *** < 0.0001***	− *4.07* *** < 0.0001***	− *3.27* *** < 0.0001***

### Preliminary analysis stratified by turn strategy

50% of pre-planned turns in this dataset were step or spin turns, but 73% of turns were step turns during late-cued turns^2^. Therefore, we completed a preliminary analysis stratifying by turn strategy. Turn strategy (step or spin) was found to significantly moderate the relationship between the study outcomes and gait phase within the two turn study conditions (pre-planned and late-cued turns, see Supplemental Document 2 for details), so the group-level statistical results are also presented stratified by turn strategy (Tables 4, 5).

**Table 4:** Group-level estimated marginal means for linear study outcomes from linear mixed models by turn strategy. Acronyms: Left Double Support (LDS) Left Single Support (LSS), Right Double Support (RDS), Right Single Support (RSS), change in global X-axis linear momentum (∆p_x_), and global X-axis average force (F_x,avg_). Negative values are leftward in the direction of the turn. Bolded p-values indicate statistically significant differences. Italic text indicates comparisons that were not related to the right single support phase that was pertinent to the hypothesis.

		Estimated marginal mean (95% CI)				Post-hoc comparisons					
	Task	LDS	LSS	RDS	RSS	LDS v. LSS	LDS v. RDS	LDS v. RSS	LSS v. RDS	LSS v. RSS	RDS v. RSS
*Spin turns*											
∆p_x_ (kg m/s)	Pre-planned	− 13.21 (− 17.82, − 8.60)	− 17.59 (− 23.54, − 11.65)	− 7.99 (− 9.37, − 6.61)	− 27.59 (− 38.60, − 16.57)	*0.49* *0.249*	− *0.97* *0.102*	1.07 0.077	− *1.27* ***0.013***	0.69 0.231	1.56 **0.0048**
	Late-cued	− 10.82 (− 14.62, − 7.01)	− 26.74 (− 37.30, − 16.17)	− 6.48 (− 8.71, − 4.26)	− 29.99 (− 44.70, − 15.28)	*1.10* ***0.025***	− *0.76* *0.108*	1.24 **0.043**	− *1.44* ***0.002***	0.16 0.721	1.56 **0.012**
F_x,avg_ (N)	Pre-planned	− 72.67 (− 95.62, − 49.72)	− 41.27 (− 54.85, − 49.72)	− 44.44 (− 52.06, − 36.82)	− 66.71 (− 92.06, − 41.37)	− *1.02* *0.131*	− *1.02* *0.131*	− 0.15 0.999	*0.16* *0.999*	0.76 0.327	0.73 0.327
	Late-cued	− 56.17 (− 76.26, − 36.07)	− 54.37 (− 77.13, − 31.61)	− 37.19 (− 51.90, − 22.47)	− 73.89 (-106.09, − 41.69)	− *0.05* *0.978*	− *0.64* *0.663*	0.41 0.978	− *0.48* *0.839*	0.41 0.978	0.91 0.254
*Step* *turns*											
∆p_x_ (kg m/s)	Pre-planned	− 9.71 (− 11.86, − 7.56)	− 7.64 (− 9.29, − 5.99)	− 11.17 (− 13.56, − 8.79)	− 32.41 (− 41.71, − 23.11)	− *0.43* *0.265*	*0.35* *0.366*	1.93 ** < 0.0001**	*0.68* *0.053*	2.04 ** < 0.0001**	1.78 **0.0001**
	Late-cued	− 16.22 (− 21.00, − 11.45)	− 7.15 (− 9.85, − 4.46)	− 8.08 (− 10.02, − 6.14)	− 55.39 (− 67.60, − 43.18)	− *0.94* ***0.0046***	− *1.08* ***0.0048***	2.24 ** < 0.0001**	*0.12* *0.578*	2.75 ** < 0.0001**	2.87 ** < 0.0001**
F_x,avg_ (N)	Pre-planned	− 54.67 (− 65.99, − 43.34)	− 18.02 (− 21.95, − 14.09)	− 62.49 (− 76.66, − 48.33)	− 78.61 (− 99.54, − 57.69)	− *2.18* *** < 0.0001***	*0.32* *0.415*	0.80 0.146	*2.02* *** < 0.0001***	2.15 ** < 0.0001**	0.49 0.415
	Late-cued	− 77.44 (− 98.88, − 56.00)	− 14.26 (− 20.56, − 7.96)	− 47.69 (− 59.69, − 35.69)	− 124.98 (− 152.50, − 97.47)	− *1.81* *** < 0.0001***	− *0.80* ***0.018***	1.00 **0.017**	*1.19* *** < 0.0001***	2.72 ** < 0.0001**	1.82 ** < 0.0001**

**Table 5:** Group-level estimated marginal means for angular study outcomes from linear mixed models by turn strategy. Acronyms: Left Double Support (LDS) Left Single Support (LSS), Right Double Support (RDS), Right Single Support (RSS), change in transverse-plane angular momentum (∆H_z_) and average transverse-plane moment (M_z,avg_). Positive values for H_z_ and M_z_ rotate the body leftwards, in the direction of the turn. Bolded p-values indicate statistically significant differences. Italic text indicates comparisons that were not related to the left double support phase that was pertinent to the hypothesis.

		Estimated marginal mean (95% CI)				Post-hoc comparisons Cohen’s d and p-values					
	Task	LDS	LSS	RDS	RSS	LDS v. LSS	LDS v. RDS	LDS v. RSS	LSS v. RDS	LSS v. RSS	RDS v. RSS
*Spin turns*											
∆H_z_ (kg m^2^/s)	Pre-planned	2.65 (1.80, 3.49)	− 1.42 (− 1.86, − 0.99)	− 2.42 (− 2.85, − 1.98)	1.46 (1.16, 1.76)	4.08 ** < 0.0001**	5.13 ** < 0.0001**	1.23 **0.0102**	*1.48* ***0.004***	*−* *4.48* *** < 0.0001***	*−* *6.17* *** < 0.0001***
	Late-cued	2.09 (1.72, 2.47)	− 1.53 (− 2.14, − 0.92)	− 1.70 (− 2.14, − 1.27)	1.51 (0.84, 2.18)	5.30 ** < 0.0001**	4.65 ** < 0.0001**	0.76 0.270	*0.19* *0.652*	*−* *3.61* *** < 0.0001***	*−* *3.25* *** < 0.0001***
M_z,avg_ (Nm)	Pre-planned	14.41 (10.42, 18.40)	− 3.26 (− 4.18, − 2.34)	− 13.50 (− 16.07, − 10.92)	3.53 (2.71, 4.34)	3.92 ** < 0.0001**	5.35 ** < 0.0001**	2.42 ** < 0.0001**	*3.35* *** < 0.0001***	*−* *4.39* *** < 0.0001***	*−* *11.19* *** < 0.0001***
	Late-cued	11.27 (9.06, 13.49)	− 3.43 (− 4.68, − 2.18)	− 9.62 (− 12.23, − 7.01)	3.60 (2.08, 5.12)	5.06 ** < 0.0001**	4.82 ** < 0.0001**	2.53 ** < 0.0001**	*1.69* *** < 0.0001***	*−* *3.60* *** < 0.0001***	*−* *3.51* *** < 0.0001***
*Step* *turns*											
∆H_z_ (kg m^2^/s)	Pre-planned	2.45 (1.78, 3.12)	− 1.32 (− 1.60, − 1.03)	− 2.25 (− 2.65, − 1.84)	1.21 (0.93, 1.50)	4.52 ** < 0.0001**	4.81 ** < 0.0001**	1.48 **0.001**	*1.38* ***0.0007***	*−* *5.63* *** < 0.0001***	*−* *5.10* *** < 0.0001***
	Late-cued	2.22 (1.65, 2.79)	− 1.70 (− 2.06, − 1.34)	− 1.07 (− 1.26, − 0.89)	0.98 (0.71, 1.26)	4.48 ** < 0.0001**	3.92 ** < 0.0001**	1.42 **0.0005**	*−* *1.05* ***0.003***	*−* *4.23* *** < 0.0001***	*−* *3.52* *** < 0.0001***
M_z,avg_ (Nm)	Pre-planned	13.32 (9.98, 16.65)	− 3.14 (− 3.82, − 2.45)	− 12.32 (− 14.54, − 10.09)	2.84 (2.07, 3.61)	4.11 ** < 0.0001**	4.67 ** < 0.0001**	2.61 ** < 0.0001**	*2.35* *** < 0.0001***	*−* *5.42* *** < 0.0001***	*−* *3.88* *** < 0.0001***
	Late-cued	10.86 (8.20, 13.53)	− 4.11 (− 4.95, − 3.28)	− 6.56 (− 7.96, − 5.17)	2.20 (1.59, 2.81)	4.11 ** < 0.0001**	3.95 ** < 0.0001**	2.39 ** < 0.0001**	*0.85* ***0.004***	*−* *4.40* *** < 0.0001***	*−* *3.13* *** < 0.0001***

When stratified by turn strategy, the unstratified rotational group trends are mostly still present: left double support was the gait phase that generated significantly greater ∆H_z_ and greater ${\mathrm{M}}_{{\mathrm{z}}_{\mathrm{avg}}}$ (p-values ≤ 0.0102) in each step and spin turns, with the exception that during late-cued spin turns, the ∆H_z_ during left double support was not significantly different vs. right single support (p = 0.270; Table 5).

When stratified by turn strategy, the linear findings were consistent with the unstratified group trends during step turns but were not during spin turns (Table 4). During pre-planned step turns**,** leftward ∆p_x_ was greater during right single support vs. any other phase and leftward F_x,avg_ was greater during right single support vs. left single support (p < 0.0001 for each comparison), which were the unstratified group trends. During late-cued step turns, leftward ∆p_x_ and F_x,avg_ were greater during right single support vs. any other phase (p-values ≤ 0.017), which was the unstratified group trend. However, during spin turns, the linear findings were not consistent with the unstratified group trends. During spin turns, leftward ∆p_x_ was greater during right single support vs. right double support during pre-planned and late-cued turns (p = 0.0048 and p = 0.012, respectively) and was greater during right single support vs. left double support during late-cued turns (p = 0.043) but was not different during right single support vs. left double support during pre-planned turns (p = 0.077). During pre-planned and late-cued spin turns, leftward F_x,avg_ was not significantly different during right single support vs. any other gait phase (p-values ≥ 0.254).

**Figure 7:**
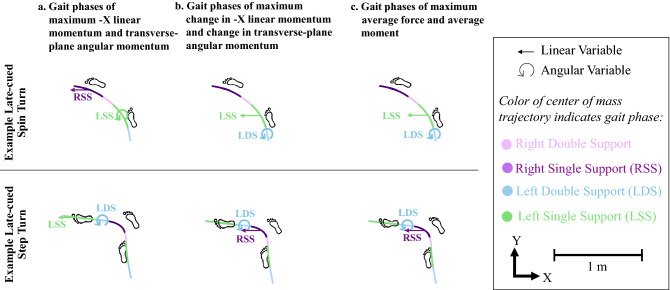
Two example late-cued turn trials classified as a spin turn (top) and a step turn (bottom) performed by the same participant with indications of the gait phases that generated the largest (**a**) − X linear and transverse-plane angular momentum, (**b**) change in − X linear and transverse-plane angular momentum, (**c**) average − X linear force and average moment about vertical. During the spin turn, the left single support generated the largest change in COM leftward momentum and largest average leftward (− X) linear force. During the step turn, the right single support generated the largest change in leftward (− X) force and largest average COM leftward linear acceleration. Additionally, during both the step turn and the spin turn, the left double support (double support with the left foot in front) generated the largest change in transverse-plane angular momentum and largest average moment about vertical.

Figure 7 illustrates two example late-cued trials that demonstrate that the greatest leftward ∆p_x_ (Fig. 7b) and greatest leftward F_x,avg_ (Fig. 7c) often occurred during left double or left single support (see estimated marginal means in Table 4), but during step turns, these linear variables were the greatest during right single support, which was consistent with the primary unstratified study group trends. These examples do not represent a unanimous strategy used in all trials, but they do illustrate common patterns observed during spin turns.

### Between-task comparison within gait-phase

Though there were no a priori hypotheses about how these variables change across tasks, the specification of the linear mixed models allowed exploration into whether the variables of interest in each gait phase differed by task (Table 6). Within the left double support phase, which was the phase of interest for ∆H_z_ and M_z,avg_, there were no significant differences between tasks (p-values ≥ 0.753). Additionally, during right double support, ∆H_z_ and M_z,avg_ were significantly greater (values were less negative) during late-cued turns vs. straight line gait and pre-planned turns (p-values ≤ 0.0003). Within the right single support phase, which was the phase of interest for change in leftward ∆p_x_ and average leftward F_x,avg_, there were significant differences between each task such that these variables were largest during late-cued turns and smallest during straight-line gait (p-values ≤ 0.019). Additionally, these variables were significantly larger during turns vs. straight line gait within each gait phases (p-values ≤ 0.002).

**Table 6:** Linear and angular study outcomes comparisons across task within-gait phase from linear mixed models. Acronyms: Left Double Support (LDS) Left Single Support (LSS), Right Double Support (RDS), Right Single Support (RSS), change in transverse-plane angular momentum (∆H_z_) average transverse-plane moment (M_z,avg_), linear momentum (∆p_x_), and global X-axis average force (F_x,avg_). Positive values for H_z_ and M_z_ rotate the body leftwards, in the direction of the turn. Negative values for p_x_ and F_x_ are leftward in the direction of the turn. Bolded p-values indicate statistically significant differences. Italic text indicates comparisons that were not related to the gait phase that was pertinent to the linear or angular hypotheses. Note, estimated marginal means and confidence intervals for these study outcomes have already been provided in Tables 1 and 2.

		Cohen’s D and p-values			
	Task	LDS	LSS	RDS	RSS
M_z,avg_ (Nm)	Straight-line gait v. late-cued turns	0.37 p = 0.753	*0.62* *p = 0.359*	*−* *1.54* ***p = 0.0001***	*0.70* *p = 0.165*
	Straight-line gait v. pre-planned turns	− 0.18 p = 0.753	*0.01* *p = 0.988*	*−* *0.21* *p = 0.597*	*0.36* *p = 0.475*
	Late-cued v. pre-planned turns	− 0.48 p = 0.753	*−* *0.57* *0.359*	*1.23* ***p = 0.0003***	*−* *0.38* *p = 0.475*
∆H_z_ (kg m^2^/s)	Straight-line gait v. late-cued turns	0.03 p = 0.999	*0.69* *p = 0.255*	*−* *1.60* *** < 0.0001***	*0.59* *p = 0.282*
	Straight-line gait v. pre-planned turns	− 0.23 p = 0.999	*0.15* *p = 0.730*	*−* *0.20* *p = 0.645*	*0.26* *p = 0.536*
	Late-cued v. pre-planned turns	− 0.25 p = 0.999	*−* *0.50* *p = 0.421*	*1.53* ***p < 0.0001***	*−* *0.38* *p = 0.456*
F_x,avg_ (N)	Straight-line gait v. late-cued turns	*2.36* ***p < 0.0001***	*2.30* ***p < 0.0001***	*2.21* ***p < 0.0001***	2.03 **p < 0.0001**
	Straight-line gait v. pre-planned turns	*2.70* ***p < 0.0001***	*3.47* ***p < 0.0001***	*3.16* ***p < 0.0001***	1.33 **p = 0.001**
	Late-cued v. pre-planned turns	*−* *0.34* *p = 0.347*	*0.10* *p = 0.603*	*0.38* *p = 0.178*	− 0.88 **p = 0.019**
∆p_x_ (kg m/s)	Straight-line gait v. late-cued turns	*2.26* ***p < 0.0001***	*2.33* ***p < 0.0001***	*2.32* ***p < 0.0001***	2.12 ** < 0.0001**
	Straight-line gait v. pre-planned turns	*2.53* ***p < 0.0001***	*3.30* ***p < 0.0001***	*3.27* ***p < 0.0001***	1.31 **p = 0.002**
	Late-cued v. pre-planned turns	*−* *0.58* *p = 0.112*	*−* *0.05* *p = 0.819*	*0.53* *p = 0.068*	− 1.02 **p = 0.009**

## Discussion

This study focused on understanding the gait-phase specific generation of global leftward linear and angular momenta during straight-line gait, pre-planned, and late-cued 90° leftward turns. Partially supporting our first hypothesis, we found a significantly greater change in leftward linear momentum (analogous to normalized linear impulse) during right single support phase vs. any other gait phase during all three tasks. Additionally, during straight-line and late-cued turns, the average leftward force was significantly greater during right single support vs. any other phase. However, during pre-planned turns, average leftward force during right single support was only significantly greater than it was during left single support. Supporting our second hypothesis, we found a significantly greater leftward change in transverse-plane angular momentum (analogous to angular impulse) and greater average transverse-plane moment during the double support phase with the left foot in front (left double support) vs. any other gait phase during all three tasks.

These results suggest that during turns, changes in body-facing direction leftward are initiated most during the left double support and that changes in COM leftward linear momentum occur more during right single support phase. This occurred despite a diversity in the number of footfalls and turn strategies used to execute the turn. This behavior during turns aligns with the understanding that during straight-line gait, the change in linear momentum is leftward during right single support phases^24^ and that the change in transverse-plane angular momentum is leftward during left double support^30^. This alignment provides emerging evidence of momenta control strategies that are shared across straight-line and turning gait.

During the right single support, the body generates the greatest leftward COM linear impulse. To review, during straight-line gait, the right foot's single support phase generates a leftward linear impulse (change in linear momentum), which facilitates the center of mass translating towards the left foot^24^. This leftward linear impulse is matched with an (approximately) equal and opposite rightward linear impulse during left single support because the goal is to maintain net zero change in right-left linear momentum over repeating gait cycles. During 90° turns to the left, the goal is to generate leftward linear momentum, until the COM trajectory is in the new desired direction of travel. In this study, right single support generated approximately two to four times greater leftward linear impulse during pre-planned and late-cued turns, respectively, vs. straight-line gait. During turns, the other three gait phases also generated leftward linear impulse, in agreement with medially-directed impulses reported during circular gait^22,26^. This study uncovers that the right single support is the dominant phase for generating leftward linear momenta in straight line gait and turns performed by healthy young adults.

During the left double support, the body generates the greatest transverse-plane leftward angular impulse and greatest average moment about vertical vs. the other gait phases. To review, during straight-line gait, the double support phase with the left foot in front generates the greatest leftward angular impulse (positive change in transverse-plane angular momentum)^30^, which facilitates the following right leg swing phase. This angular impulse is matched with an (approximately) equal and opposite change in angular momentum towards the right during right double support because the goal in straight-line gait is to maintain net zero transverse-plane angular momentum over large numbers of gait cycles. In contrast, during leftward turns, the goal is to generate additional leftward transverse-plane angular momentum to initiate changing the body’s facing direction (e.g., by ~ 90° during a 90° turn), then arrest this additional angular momentum when the desired facing direction of the body is achieved. We found that during straight-line gait and turns, the greatest transverse-plane leftward moment was applied during left double support. This is reasonable, as both feet can coordinate to generate rotation while maintaining balance^16,17,34^. When both feet are in contact with the ground, not only is the base of support larger which may facilitate balance maintenance, but ground reaction forces can apply a moment without applying extraneous linear impulse that can adversely affect balance or COM linear trajectory goals. In other words, the linear impulse generated by each leg’s transverse-plane ground reaction forces can cancel across legs in directions of unwanted linear momentum, though each force can work together apply a moment about a vertical axis passing through the COM and linear impulse in the desired direction of travel (e.g., illustrative example in Fig. 1)^16,17,34^.

Though comparing across tasks was not the central research question for this paper, there are important contrasts to share from the within-gait phase, across task analysis. As reviewed, the patterns of the greatest leftward linear impulse generated during right single support and the greatest transverse-plane leftward angular impulse generated during left double support were similar within each task of straight-line gait, pre-planned, and late-cued turns. In a post-hoc analysis, we found that angular momentum generating variables within left double support did not differ across task, but leftward linear momentum variables were largest during late-cued turns compared to during pre-planned turns and straight-line gait. During turn conditions, all four gait phases contribute to leftward *linear* impulse, differing from straight-line gait. However, how each gait phase contributes to leftward *angular* impulse is similar across the tasks of straight-line gait, pre-planned turns, and late-cued turns. Though future research can answer why we observed this difference in linear and angular momenta control, we suspect that this can be attributed to the following contexts that may limit the generation of leftward body rotation to primarily be the left double support phase. For one, the demand to rotate the body’s facing direction is transient, while the need to generate and sustain a leftward linear momentum is maintained throughout the turn phase as the person continues to walk in the new direction. Second, maintaining balance while generating leftward body rotation about a vertical axis is facilitated by doing so during double support, when the base of support is larger than single support and when both legs can mitigate extraneous momentum. Third, rotating the body towards the left prior to left single support can facilitate the transition to the right lower extremity swinging forward towards its next footfall (right swing phase). The second and third contexts can limit the opportunity to generate leftward rotation of the body primarily to the left double support phase.

Within each turn condition, participants used a variety of footfalls that led to identifying both “step” and “spin” turn strategies, which is likely representative of what is used in daily life. In spin turns, the inside (left) foot was closest to the center of the intersection, and vice-versa for step turns^37^. When we performed a preliminary analysis stratified by turn strategy, we found that during spin turns, there were different momenta generating patterns compared to the unstratified analysis (step turns were similar to unstratified analysis). We offer possible explanations to be further explored in future studies. Spin turns were identified when the left (inside) foot was closest to the middle of the intersection. During spin turns, we found that these participants leaned such that their COM was often leftward of the left foot base of support (as in Figs. 2a, 7)^2^. This can relate to the finding in this study that right single support during spin turns did not generate significantly greater changes in average leftward force vs. left double support or left single support (in fact, during pre-planned spin turns, the estimated marginal mean of average leftward force during left double support exceeded that of right single support). When the COM traverses left of the left foot’s base of support, as it did more often during spin turns, the left single and double support phases can generate greater leftward linear momenta. Additionally, during late-cued turns, we found fewer (two to four) footfalls vs. three to five footfalls used in pre-planned turns^2^. With fewer gait phases, during spin turns, the left single and double support phases could greatly contribute to generating leftward linear momenta due to the opportunity presented by the left foot’s placement within the center of the intersection (Fig. 7). In contrast, when the right foot was closest to the middle of the intersection during late-cued step turns (as in Fig. 2b, 7), the right single support can still be used as the primary gait phase to generate leftward linear momentum. Future research should be conducted to explore these possibilities. A limitation of our analysis stratifying by turn type includes that we observed 27% spin turns during late-cued turns, which could be limiting statistical power in comparing across gait phases within the spin turn strategy of late-cued turns. Thus, this stratified analysis and interpretation are considered preliminary.

This study has several limitations to note. First, although we expect left and right turns to be controlled similarly, we only examined left turns. We administered a visual late cue to turn, but future research can uncover how this compares to auditory cues to turn. It is unknown how turning the head towards a visual cue prior to the turn phase affects the overall transverse-plane leftward angular impulse generated during the turn phase. Additionally, even though this study leveraged within-subject statistical designs, this study did not normalize variables by mass. Thus, different trends may be determined when accounting for differing participant masses, particularly for linear momentum variables that require accelerating a mass through space (vs. angular variables that rotate the body about its COM). This study used blocks from straight-line gait to pre-planned and late-cued turns, but future research should randomize these blocks or trials. Also, our turn phase definition relied on pelvis rotation, when other preparatory adjustments may have preceded pelvis rotation, such as rotation of other body segments (like the head) or changes in the COM trajectory. Relatedly, although we intended to explore how turns are initiated, our turn phase may consist of both turn initiation and termination, which may include arresting transverse-plane angular momenta generated earlier. Future research can compare how turn phases are defined and further parsed into sub-phases (initiation and termination, for example) and can identify if these momenta generation patterns differ during specific gait phases that occur early vs. late in the turn phase, or if they differ if a particular gait phase does not occur during the turn phase. Finally, we plan to expand our sample to include more females because this sample only included three females and was therefore too small to investigate if there were differences in the studied relationships between sexes.

This study opens many future research questions. We would like to understand how the generation of transverse plane momenta relates to frontal-plane balance maintenance^2^ and how these momenta generation strategies are used by different balance-impaired populations. For instance, how will momenta generation strategies differ across different populations like people with Parkinson’s disease, who may turn “en-bloc” (body segments rotating concurrently)?^38^ Future research will investigate temporal patterns in momenta control, for example, if generating the requisite momenta tended to occur in a sequence (e.g., rotation prior to translation). By more fully understanding the role of each gait phase towards the required generation of momenta during different types of turns, we can better assist people who struggle to balance during turns.

In summary, when young adults performed straight-line gait, pre-planned and late-cued turns, they exhibited similar momenta generation patterns between gait phases, especially in the generation of transverse-plane angular momenta. Specifically, in straight-line gait and turns, the greatest change in linear momentum towards the new direction of travel occurred during right single support. Additionally, during straight-line gait and turns, the greatest change in transverse-plane angular momentum and average moment to rotate the body to the left occurred during left double support. These patterns suggest that healthy young adults leverage straight-line gait momenta generation strategies during pre-planned and late-cued 90° turns.

## Methods

### Participants, experimental setup, and procedures

Ten healthy adults (3 females, 7 males; 25.2 ± 4.2 years; 73.9 ± 14.8 kg; 1.79 ± 0.1 m) provided informed consent to voluntarily participate in this study. The protocol was approved by the Stevens Institute of Technology Institutional Review Board and carried out in accordance with relevant guidelines, regulations, and the Declaration of Helsinki. Informed consent was obtained from all participants. The participants were included if they were between the ages of 18–64 and stated that they did not fall in the past half-year, and that they did not have a medical condition or pain that would restrict their ability to walk and turn during everyday activities.

To replicate conditions in a grocery store, tape on the floor mimicked two aisles (each 0.91 m wide) to form a perpendicular intersection (illustration included in Supplemental Document 2). Retroreflective markers placed on participants measured kinematics with optical motion capture (200 fps, Motive 2.2, NaturalPoint, Corvallis, OR, USA). Markers were placed atop the following anatomic landmarks: sternum jugular notch, sternum xiphoid process, C7, T2, and T7 vertebrae, as well as left and right: anterior and posterior head, glenohumeral joint, clavicle- acromion joint, humerus lateral epicondyle, posterior aspect of the upper arm, radial and ulnar styloid processes, second and fourth metacarpal, anterior and posterior superior iliac spines, femoral greater trochanter, anterior aspect of the thigh, femoral lateral epicondyle, fibular attachment to the tibia, tibial tuberosity, anterior aspect of the shank, lateral malleolus, first and fifth metatarsal, first distal phalanx, and calcaneus.

Participants pretended that they were in a grocery store walking at a comfortable pace in three contexts: walking straight, pre-planned turns, and late-cued turns (see Supplemental Video for example first-person view of the turn tasks). They were instructed to walk as if they were walking with people behind them (so that they should not stop suddenly), but they were not in a rush. First, they performed five trials of straight-line gait down the 10 m aisle. Next, they performed 10 pre-planned turns, and finally 10 late-cued turns to the left. Due to time constraints, only leftward turns were performed. Between trials, 15 s rest periods were provided. Prior to each of the three condition blocks, five-minute instructional and practice periods were provided. For both turn conditions, we randomly prescribed which foot to use to start walking. During the pre-planned turns, participants were instructed ahead of time that they should turn 90° left because the aisle contained the item of interest, as though they were familiar with its location in this grocery store. A large television monitor (2.03 m diagonal) at the end of the intersecting aisle displayed a large image of the item of interest, green broccoli. In the late-cued turn condition, participants knew there was a 50% chance they needed to turn left into the aisle, as though they were unfamiliar with this store and needed to look down the aisle to determine whether to turn or continue straight. When the participants reached the intersection, the monitor would display either the green broccoli to cue a turn, or a “NO” symbol (red circle with a line through it) to cue continuing to walk straight. Thus, 10 late-cued turns and 10 catch trials (continue straight-line gait) were performed. Catch trials were not included in this analysis.

### Kinematic analyses

Marker data were gap-filled and smoothed with a cubic spline filter (MATLAB ‘csaps’ function; smoothing value at 0.0005). Four trials from two subjects were excluded because of missing data due to marker occlusion. Marker data allowed generation of a model of each participant following scaled segment parameters^39,40^, a computed shoulder joint center offset from the acromion^41^, and a computed hip joint center from a calibration movement trial^42^. Turn strategy was classified for a preliminary analysis using a method such that a left turn with the right (outside) foot closest to the middle of the intersection was labeled a “step turn”, whereas, turns with the left (inside) foot closest to the middle of the intersection was labeled a “spin turn”^2,37^.

### Phases of interest

The phase of interest during straight-line gait trials was when the COM was within the center 6 m of the walkway, bounded by heel-strike events. During turn trials, the “turn phase” was the phase of interest (Fig. 2). The turn phase was defined by a person-specific threshold of the pelvis heading angle. The threshold used three standard deviations from the mean during straight-line gait trials. The start of the turn phase was the heel strike before the pelvis threshold was reached. The end of the turn phase was the first heel strike after the pelvis heading angle reduced below the threshold relative to the new direction of travel (-X direction, Fig. 2).

### Gait phases

Heel strike and toe-off gait events were detected using the relative positioning of the foot markers and pelvis^43^ modified for turning gait^44^. Left double support phase is the interval from left heel strike to the frame before right toe off, and vice versa for right double support. Left single support is the interval from right toe off to the frame before right heel-strike, and vice versa for right single support. We performed all analyses regarding transverse-plane angular and linear momenta within each of the four phases of gait (left and right single and double support).

### Transverse-plane angular momentum (H_z_) and average moment (M_z,avg_)

Whole-body angular momentum about the COM was computed and normalized using methods previously described^45^. Briefly, angular momentum is computed as the sum of each body segment’s “local” and “remote” angular momentum terms at each timepoint. The local angular momentum term is the product of the body segment’s inertial tensor and its angular velocity with respect to the global coordinate system. The remote angular momentum term is the cross product of the position vector (from the whole-body COM to the individual body segment’s COM) and the linear momentum of that body segment with respect to the global coordinate system^45^. Angular momentum about the vertical axis through the COM provided transverse-plane angular momentum (H_z_). Positive H_z_ rotates the body leftward, towards the new body facing direction (Fig. 2). We computed the change in Hz during each of the four gait phases, which provides insight about angular impulse generation. During each gait phase, we also computed the normalized average moment about vertical (change in Hz divided by phase duration divided by body mass) to compare across gait phases.

### Global X-axis linear momentum (p_x_) and average force (F_x_)

Horizontal linear momentum in the direction of the turn was computed along the global X-axis (leftward is negative) as the X-component of the COM velocity multiplied by the participant’s mass (Fig. 2). We computed the change in leftward linear momentum during each of the four gait phases. We also computed the average leftward average force to compare across gait phases.

### Statistical analyses

Differences in study outcomes (average acceleration, average moment, change in linear velocity, and change in angular momentum) across gait phases (right double support, right single support, left double support, left single support) within study task (straight-line gait, pre-planned and late-cued turn conditions) and across task within gait phase were examined using linear mixed models (LMMs). The LMMs included fixed effects (or main factors) for gait phase, study task, and a gait phase by study task interaction. Additionally, the models included random intercepts for study participant and random slopes for trial number nested within study task and study participant (*proc* *glimmix* in SAS version 9.4; SAS Institute Inc., Cary, NC). The general form of the models was estimated and their overall results are provided in Supplemental Document 2. If there were multiple instances of a gait phase within the phase of interest (e.g. Fig. 2a, right single support), the outcome variables were averaged across the multiple instances within that trial. The number of repetitions of gait phases ranged from 3 to 5 during straight-line gait, 1–2 during pre-planned turns, and 0–2 during late-cued turns. If there were no instances of a particular gait phase within a trial, the gait phase outcome variable was assigned a missing value for that trial and that trial was excluded from analyses.

Mixed models were chosen as the analytic technique because they appropriately handle repeated measurements within study participants and maximize the information among participants if data are missing. Pairwise comparisons of the study outcomes between gait phases within each study task and between tasks within each gait phase were conducted within the regression models via orthogonal contrasts (*lsmeans* function). The Holm test was used to correct for multiple comparisons and to maintain a two-tailed familywise alpha at 0.05 across the hypotheses tested. An adjusted p-value < 0.05 was used to determine statistical significance^46^.

A secondary analysis explored whether turn strategy (“spin” versus “step” turn) moderated the relationship between gait phase and study outcomes within each study condition. To do this, a three-way interaction term between gait phase, study condition, and turn strategy was added to the above-described linear mixed models. Since straight-line gait has no associated turn strategy, this analysis was limited to the subset of observations from pre-planned and late-cued turns. A p-value < 0.05 for the three-way interaction indicated that moderation effects were present. Due to the moderation effects that were present, results are also presented stratified by turn strategy.

### Within-participant statistical analysis

Linear mixed models were used to explore differences in the study outcomes across gait phases within study task for each study participant separately. This analysis was conducted to determine how many participants exhibited the group trends. The model included fixed factors for gait phase, study task, and gait phase X study task, as well as a random slope for trial nested within study task. Pairwise comparisons between gait phases within each study task were conducted within the regression models via orthogonal contrasts (*lsmeans* function). The Holm test was used to correct for multiple comparisons and to maintain a two-tailed familywise alpha at 0.05 across the hypotheses tested. These results are included in Supplemental Document 2.

### Sample size justification

Power was computed using PASS 2023, version 23.0.1. The primary outcomes of interest in this study were ∆H_z_, M_z,avg_, ∆p_x_, and F_x,avg_. Power was estimated assuming a hierarchical study design with a within subject correlation of 0.75 and a modeling approach of linear mixed models with post hoc comparisons to compare group means. Estimation was conducted with intent to detect a 1.25 standard deviation difference between gait phases (i.e., LDS, LSS, RDS, and RSS) within study task (i.e., straight-line gait, pre-planned turns, and late-cued turns) and between tasks within gait phases, while accommodating the Bonferroni adjusted per-comparison alpha (p < 0.0017) necessary to maintain an overall two-tailed alpha of 0.05 across the hypotheses we tested. A Bonferroni adjustment was chosen for the purposes of the power analysis because it is highly conservative and the Holm test, which will be used in all analyses, is based on the empirical p-value attained at the time of data analysis which was unavailable. Given these parameters, a sample size of 10 subjects with 5–10 observations within study task will maintain approximately 81.9% power. Additionally, this sample size will maintain 91.4% power to detect a 1.4 standard deviation difference in group means. Cohen’s D statistics are provided for each comparison to provide context of the power relative to the standard deviation difference in group means.

All de-identified data are included in Supplemental Document 1 to allow replication of the data analysis.

## Supplementary Information

Supplementary Information 1.

Supplementary Information 2.

Supplementary Video 1.

## Data Availability

All data generated or analysed during this study are included in this published article (and its Supplementary Information files).

## References

[1] Glaister BC, Bernatz GC, Klute GK, Orendurff MS (2007). Video task analysis of turning during activities of daily living. Gait Posture.

[2] Tillman M, Molino J, Zaferiou AM (2022). Frontal plane balance during pre-planned and late-cued 90 degree turns while walking. J. Biomech..

[3] Mellone S, Mancini M, King LA, Horak FB, Chiari L (2016). The quality of turning in Parkinson’s disease: A compensatory strategy to prevent postural instability?. J. Neuroeng. Rehabil..

[4] Mancini M (2015). Continuous monitoring of turning in Parkinson’s disease: Rehabilitation potential. NeuroRehabilitation.

[5] Spildooren J (2013). Head-pelvis coupling is increased during turning in patients with Parkinson’s disease and freezing of gait. Mov. Disord..

[6] Mancini M (2016). Continuous monitoring of turning mobility and its association to falls and cognitive function: A pilot study. J. Gerontol. Ser. A Biol. Sci. Med. Sci..

[7] Taylor MJD, Dabnichki P, Strike SC (2005). A three-dimensional biomechanical comparison between turning strategies during the stance phase of walking. Hum. Mov. Sci..

[8] He C (2018). Dynamic stability and spatiotemporal parameters during turning in healthy young adults. Biomed. Eng. Online.

[9] Mari S (2012). Turning strategies in patients with cerebellar ataxia. Exp. Brain Res..

[10] Hase K, Stein RB (1999). Turning strategies during human walking. J. Neurophysiol..

[11] Bonnyaud C, Roche N, Van Hamme A, Bensmail D, Pradon D (2016). Locomotor trajectories of stroke patients during oriented gait and turning. PLoS ONE.

[12] Qiao M, Feld JA, Franz JR (2018). Aging effects on leg joint variability during walking with balance perturbations. Gait Posture.

[13] Hof AL, Gazendam MGJ, Sinke WE (2005). The condition for dynamic stability. J. Biomech..

[14] Golyski PR, Vazquez E, Leestma JK, Sawicki GS (2022). Onset timing of treadmill belt perturbations influences stability during walking. J. Biomech..

[15] Bauby CE, Kuo AD (2000). Active control of lateral balance in human walking. J. Biomech..

[16] Zaferiou AM, Wilcox RR, McNitt-Gray JL (2016). Modification of impulse generation during pirouette turns with increased rotational demands. J. Appl. Biomech..

[17] Peterson TJ, Wilcox RR, McNitt-Gray JL (2016). Angular impulse and balance regulation during the golf swing. J. Appl. Biomech..

[18] Harper, S. A., Beethe, A. Z., Dakin, C. J. & Bolton, D. A. E. Promoting generalized learning in balance recovery interventions. *Brain**Sci.* **11**, NA (2021). 10.3390/brainsci11030402 PMC8004641 33810159

[19] Patla, A. E., Frank, J. S. & Winter, D. A. Balance control in the elderly: Implications for clinical assessment and rehabilitation. *Can.**J.**Public**Health.* **83**, (1992). 1468046

[20] Li P, Yamada Y, Yamada K, Yokoya M (2022). Functional resistance training with gait phase-dependent control using a robotic walker: A pilot study. IEEE Access.

[21] Desmet DM, Cusumano JP, Dingwell JB (2022). Adaptive multi-objective control explains how humans make lateral maneuvers while walking. PLoS Comput. Biol..

[22] Orendurff MS (2006). The kinematics and kinetics of turning: limb asymmetries associated with walking a circular path. Gait Posture.

[23] Glaister BC, Orendurff MS, Schoen JA, Bernatz GC, Klute GK (2008). Ground reaction forces and impulses during a transient turning maneuver. J. Biomech..

[24] Bruijn, S. M. & Van Dieën, J. H. Control of human gait stability through foot placement. *J.**R.**Soc.**Interface* **15**, (2018). 10.1098/rsif.2017.0816 PMC6030625 29875279

[25] Simoneau GG, Krebs DE (2000). Whole-body momentum during gait: A preliminary study of non-fallers and frequent fallers. J. Appl. Biomech..

[26] Glaister, B. C., Orendurff, M. S., Schoen, J. A. & Klute, G. K. Rotating horizontal ground reaction forces to the body path of progression. *J.**Biomech.* **40**, 3527–3532 (2007). 10.1016/j.jbiomech.2007.05.014 17597134

[27] Dixon PC, Stebbins J, Theologis T, Zavatsky AB (2014). Ground reaction forces and lower-limb joint kinetics of turning gait in typically developing children. J. Biomech..

[28] Strike SC, Taylor MJD (2009). The temporal-spatial and ground reaction impulses of turning gait: Is turning symmetrical?. Gait Posture.

[29] Neptune RR, Vistamehr A (2019). Dynamic balance during human movement: Measurement and control mechanisms. J. Biomech. Eng..

[30] Herr H, Popovic M (2008). Angular momentum in human walking. J. Exp. Biol..

[31] Farrell, M. T. & Herr, H. Angular momentum primitives for human turning: Control implications for biped robots. In *2008**8th**IEEE-RAS**Int.**Conf.**Humanoid**Robot.**Humanoids**2008* 163–167 (2008). 10.1109/ICHR.2008.4755962

[32] Nolasco LA, Silverman AK, Gates DH (2019). Whole-body and segment angular momentum during 90-degree turns. Gait Posture.

[33] Zaferiou AM, Wilcox RR, McNitt-Gray JL (2016). Modification of impulse generation during piqué turns with increased rotational demands. Hum. Mov. Sci..

[34] Liu JM (2022). Roles of each leg in impulse generation in professional baseball pitchers: Preliminary findings uncover the contribution of the back leg towards whole-body rotation. Sport. Biomech..

[35] Havens KL, Sigward SM (2015). Whole body mechanics differ among running and cutting maneuvers in skilled athletes. Gait Posture.

[36] Jindrich DL, Besier TF, Lloyd DG (2006). A hypothesis for the function of braking forces during running turns. J. Biomech..

[37] Golyski PR, Hendershot BD (2017). A computational algorithm for classifying step and spin turns using pelvic center of mass trajectory and foot position. J. Biomech..

[38] Akram S, Frank JS, Jog M (2013). Parkinson’s disease and segmental coordination during turning: I. Standing turns. Can. J. Neurol. Sci..

[39] Dumas, R., Chèze, L. & Verriest, J. P. Adjustments to McConville et al. and Young et al. body segment inertial parameters. *J.**Biomech.* **40**, 543–553 (2007). 10.1016/j.jbiomech.2006.02.013 16616757

[40] Dumas, R., Chèze, L. & Verriest, J. P. Corrigendum to ‘Adjustments to McConville et al. and Young et al. body segment inertial parameters’ [J. Biomech. (2006) in press] (DOI:10.1016/j.jbiomech.2006.02.013). *J.**Biomech.* **40**, 1651–1652 (2007). 10.1016/j.jbiomech.2006.02.013 16616757

[41] Rab G, Petuskey K, Bagley A (2002). A method for determination of upper extremity kinematics..

[42] Schwartz MH, Rozumalski A (2005). A new method for estimating joint parameters from motion data. J. Biomech..

[43] Zeni JA, Richards JG, Higginson JS (2008). Two simple methods for determining gait events during treadmill and overground walking using kinematic data. Gait Posture.

[44] Ulrich B, Santos AN, Jolles BM, Benninger DH, Favre J (2019). Gait events during turning can be detected using kinematic features originally proposed for the analysis of straight-line walking. J. Biomech..

[45] Vistamehr A, Neptune RR (2021). Differences in balance control between healthy younger and older adults during steady-state walking. J. Biomech..

[46] Westfall J, Kenny DA, Judd CM (2014). Statistical power and optimal design in experiments in which samples of participants respond to samples of stimuli. J. Exp. Psychol. Gen..

