# Supplementary material for: Gait-phase specific transverse-plane momenta generation during pre-planned and late-cued 90 degree turns while walking
Source: Sci Rep. 2023 Apr 26;13:6846. doi: 10.1038/s41598-023-33667-1 (PMC10133231; doi:10.1038/s41598-023-33667-1)
Supplement: Supplementary file 2 — Supplementary Information 2. [file 41598_2023_33667_MOESM2_ESM.pdf]

Supplemental Document 2

SD 2.1 Overhead view of experimental setting

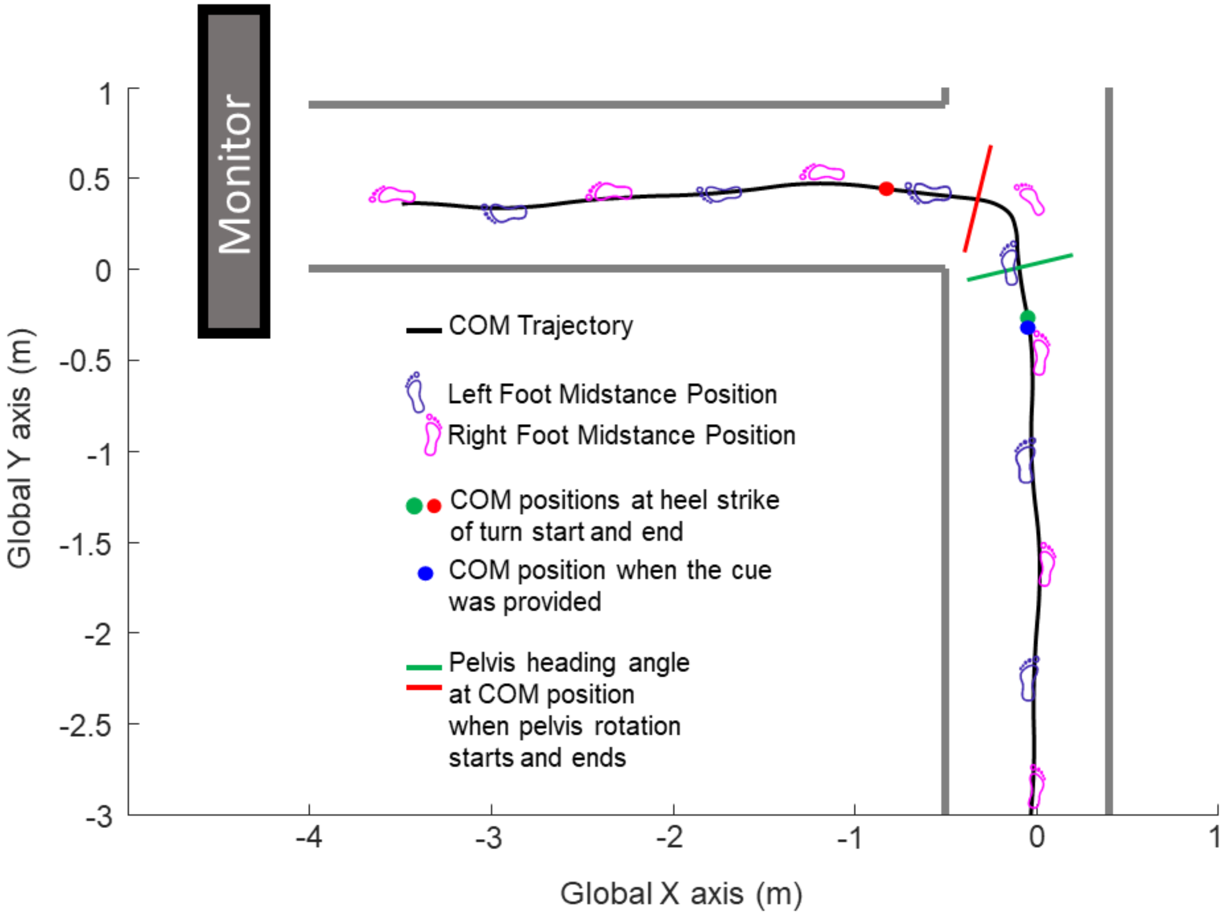

Figure SD 1. *The top-down view of the experiment setup during a late-cued turn. The participant began walking from the bottom of the image, then turned left toward the monitor after receiving the cue to turn (a green broccoli image) when they reached the intersection. Data shown are the center of mass (COM) trajectory (black line), footfall positions at midstance (blue and pink foot outlines), the COM position at start and end of the turn phase (green and red dots), pelvis orientation at the threshold values (green and red lines), and the COM position when the late cue to turn was provided (blue dot).*

SD 2.2 Linear mixed model information and results

The models for the primary analysis included fixed effects (or main factors) for gait phases and task, as well as a gait phases-by-task interaction term. Additionally, the model included a random intercept for subject and a random slope for trial. These random effects allow participant-specific estimates of the relationships between our study outcomes and gait phases and task, in addition to the marginal (or population) effects of gait phases and task. The general form of the model analyzed for each outcome is as follows:

Yij= b0 + b1jgait phasesij + b2jtaskij + b3jgait phases*taskij + ui + eij

where i represents the i-th subject, j represents the j-th trial, ui is a random intercept for subject and eij is the individual error term.

Table SD2 1 *Statistics for the original linear mixed model.*

Fixed Effect	Outcome Variable							
	ΔHz		$M_{z,avg}$		Δp_x		$F_{x,avg}$	
	**F**	**p**	**F**	**p**	**F**	**p**	**F**	**p**
**Task**	F(2, 108)=0.32	.727	F(2, 108)=0.64	.528	F(2, 108)=107.80	**<.0001**	F(2, 108)=158.09	**<.0001**
**Gait Phase**	F(3, 108)=301.02	**<.0001**	F(3, 108)=316.11	**<.0001**	F(3, 108)=32.42	**<.0001**	F(3, 108)=50.87	**<.0001**
**Task*Gait Phase**	F(6, 108)=6.43	**<.0001**	F(6, 108)=5.62	**<.0001**	F(6, 108)=12.96	**<.0001**	F(6, 108)=2.00	.072

Notice that the Task*Gait Phase interaction is statistically significant in all models, except $F_{x,avg}$. When significant, this means that the effect of gait phase on the outcomes differs by task. This finding does not necessarily mean that the overall pattern for gait phases is different for each task. Instead, it could be that the significance and direction are the same across tasks, but that the *magnitude of the differences* between gait phases varies between tasks. Due to this significant interaction effect ($p<0.05$) for three of the four outcomes, we conducted post hoc analyses for these variables of interest to further examine the effect of gait phase within each task and the effect of task within each gait phase.

Additionally, the lack of statistical significance for the task*phase interaction for $F_{x,avg}$ suggests that the effect of gait phase on $F_{x,avg}$ does not differ by task. However, given that it is close to .05, more research should be conducted to determine if this finding holds in other samples. We decided to present the results stratified by task for $F_{x,avg}$ as well because 0.072 is close to 0.05 and indicates a potential effect, even if it is weak.

In the secondary analysis that tested whether turn strategy moderated these relationships, we added the three-way interaction term gait phases*task*turn strategy along with its component parts to the above model.

Table SD2 2*. Statistics for the linear mixed models that test whether turn strategy is a moderator.*

Fixed Effect	Outcome Variable							
	Delta Hz		Avg Mz		Delta Px		Avg Fx	
	F	p	F	p	F	p	F	p
Task	F(1, 72)=0.19	.665	F(1, 72)=0.26	.610	F(1, 72)=5.83	**.018**	F(1, 72)=2.06	.155
Gait Phase	F(3, 72)=178.27	**<.0001**	F(3, 72)=185.29	**<.0001**	F(3, 72)=21.77	**<.0001**	F(3, 72)=13.22	**<.0001**
Task*Gait Phase	F(3, 72)=3.81	**.014**	F(3, 72)=3.55	**.019**	F(3, 72)=4.09	**.010**	F(3, 72)=2.32	.082
Turn Strategy	F(1, 604)=0.36	.552	F(1, 604)=0.46	.499	F(1, 604)=0.11	.739	F(1, 604)=0.02	.895
Task*Turn Strategy	F(1, 604)=0.16	.689	F(1, 604)=0.39	.535	F(1, 604)=0.62	.430	F(1, 604)=0.98	.322
Gait Phases*Turn Strategy	F(3, 604)=4.95	**.002**	F(3, 604)=6.33	**.0003**	F(3, 604)=12.07	**<.0001**	F(3, 604)=11.56	**<.0001**
Task*Gait Phases*Turn Strategy	F(3, 604)=2.56	**.054**	F(3, 604)=2.42	.065	F(3, 604)=6.23	**.0004**	F(3, 604)=4.89	**.002**

SD 2.3 Within-Participant Statistical Findings

Additional within-participant statistical analyses revealed that most participants (at least seven of ten) followed the group trends for angular variables, but in linear variables, there was more diversity across participants regarding how individuals exemplified the group trends. Within-participant measures are displayed in **Figures 3-6**

Global leftward (-X) change in linear momentum (Δp_x) and average force ($F_{x,avg}$)

During straight-line gait, all 10 participants exhibited the trends identified statistically at the group-level ("group trends") that leftward Δp_x and leftward $F_{x,avg}$ were greater during right single support vs. any other phase (p-values $\leq$ .0001). During pre-planned turns, the group trend that leftward Δp_x was greatest during right single support vs. any other gait phase was exhibited by eight participants (p-values $\leq$ .034). During pre-planned turns, the group trend that leftward $F_{x,avg}$ was greater during right single support vs. left single support was exhibited by seven participants (p-values $\leq$ .019). Three participants exhibited the hypothesized trend that $F_{x,avg}$ was greater during right single support vs. any other gait phase (p-values $\leq$ .019). During late-cued turns, the group trends that leftward Δp_x and leftward $F_{x,avg}$ were greater during right single support vs. any other phase were exhibited by eight participants (p-values $\leq$ .003) and four participants (p-values $\leq$ .003), respectively. Note, participant #6 exhibited that $F_{x,avg}$ during right double support was significantly larger than it was during right single support.

Table SD2 3 *Within-participant analysis for ΔPx (Ns) across gait phases within each task, where leftward ΔPx is negative and in the direction of the turn. Acronyms: Left Double Support (LDS) Left Single Support (LSS), Right Double Support (RDS), Right Single Support (RSS). Black text indicates hypothesis-specific gait phase comparisons. Bolded p-values indicate significant differences.*

		Estimated Marginal Mean (95% CI)				Post-hoc comparisons					
	**Task**	**LDS**	**LSS**	**RDS**	**RSS**	**LDS v. LSS**	**LDS v. RDS**	**LDS v. RSS**	**LSS v. RDS**	**LSS v. RSS**	**RDS v. RSS**
**Participant 1**	**Straight**	-0.51 (-0.99, -0.02)	10.68 (10.18, 11.18)	1.37 (1.02, 1.72)	-11.57 (-12.43, -10.70)	<.0001	<.0001	**<.0001**	<.0001	**<.0001**	**<.0001**
	**Pre-planned**	-11.22 (-12.28, -10.17)	-9.62 (-12.58, -6.66)	-10.19 (-11.85, -8.54)	-28.53 (-21.92, -25.14)	.431	.868	**<.0001**	.868	**<.0001**	**<.0001**
	**Late - Cued**	-13.20 (-18.79, -7.60)	-8.65 (-16.62, -0.68)	-4.65 (-5.96, -3.34)	-47.63 (-51.88, -43.38)	**.014**	**.014**	**<.0001**	.319	**<.0001**	**<.0001**
**Participant 2**	**Straight**	-0.44 (-0.70, -0.18)	5.65 (5.09, 6.22)	0.21 (-0.11, 0.53)	-5.52 (-6.38, -4.66)	<.0001	**.025**	**<.0001**	<.0001	**<.0001**	**<.0001**
	**Pre-planned**	-8.32 (-9.71, -6.93)	-10.33 (-11.54, -9.12)	-6.95 (-7.73, -6.17)	-15.10 (-17.50, -12.69)	**.0006**	.173	**.0003**	.001	**.004**	**<.0001**
	**Late - Cued**	-14.47 (-17.91, -11.02)	-18.25 (-29.80, -6.70)	-8.10 (-10.41, -5.79)	-35.58 (-50.59, -20.57)	.583	**.042**	**.026**	.160	.338	**.014**
**Participant 3**	**Straight**	-1.84 (-1.95, -1.72)	14.15 (13.25, 15.05)	1.42 (1.09, 1.75)	-13.53 (-14.93, -12.12)	<.0001	<.0001	**<.0001**	<.0001	**<.0001**	**<.0001**
	**Pre-planned**	-13.75 (-15.80, -11.69)	-13.81 (-16.95, -10.67)	-10.37 (-12.16, -8.57)	-32.84 (-38.66, -27.02)	.945	.231	**<.0001**	.306	**<.0001**	**<.0001**
	**Late-Cued**	-28.81 (-30.93, -26.69)	-16.39 (-20.37, -12.41)	-6.37 (-9.40, -3.34)	-63.96 (-67.10, -60.82)	<.0001	<.0001	**<.0001**	.005	**<.0001**	**<.0001**
**Participant 4**	**Straight**	-1.32 (-1.87, -0.77)	14.63 (13.93, 15.32)	-0.55 (-9.75, -0.34)	-12.22 (-13.65, -12.79)	<.0001	**.027**	**<.0001**	<.0001	**<.0001**	**<.0001**
	**Pre-planned**	-8.34 (-9.21, -7.46)	-2.96 (-5.46, -0.45)	-8.68 (-9.92, -7.44)	-19.62 (-21.06, -18.19)	<.0001	.646	**<.0001**	<.0001	**<.0001**	**<.0001**
	**Late - Cued**	-9.30 (-12.17, -6.43)	-12.64 (-21.90, -3.38)	-8.16 (-10.72, -5.59)	-32.55 (-41.90, -23.20)	.971	.971	**.0001**	.944	.133	**.0003**
**Participant 5**	**Straight**	1.47 (1.31, 1.62)	8.09 (7.65, 8.53)	-1.21 (-1.32, -1.10)	-7.56 (-8.58, -6.53)	<.0001	<.0001	**<.0001**	<.0001	**<.0001**	**<.0001**
	**Pre-planned**	-4.28 (-5.23, -3.34)	-10.47 (-16.58, -4.37)	-7.30 (-8.43, -6.18)	-24.75 (-27.91, -21.58)	**.047**	**.006**	**<.0001**	.360	**.006**	**<.0001**
	**Late-Cued**	-3.31 (-4.57, -2.06)	-4.89 (-10.81, 1.02)	-6.99 (-8.31, -5.67)	-30.28 (-35.85, -24.70)	.999	**.013**	**<.0001**	.999	**.0003**	**<.0001**
**Participant 6**	**Straight**	-0.97 (-1.23, -0.71)	12.17 (11.49, 12.85)	1.25 (1.03, 1.47)	-12.50 (-13.55, -11.46)	<.0001	<.0001	**<.0001**	<.0001	**<.0001**	**<.0001**
	**Pre-planned**	-12.05 (-13.47, -10.63)	-16.59 (-21.38, -10.63)	-8.66 (-9.73, -7.60)	-25.81 (-29.00, -22.63)	**.034**	**.023**	**<.0001**	**.023**	**.034**	**<.0001**
	**Late Cued**	-16.23 (-19.13, -13.33)	-26.17 (-39.41, -12.93)	-7.74 (-9.57, -5.92)	-54.37 (-56.13, -52.61)	.174	<.0001	**<.0001**	.020	**.0006**	**<.0001**
**Participant 7**	**Straight**	-1.02 (-1.303, -0.728)	12.76 (12.20, 13.31)	0.75 (0.42, 1.09)	-12.06 (-12.68, -11.45)	<.0001	<.0001	**<.0001**	<.0001	**<.0001**	**<.0001**
	**Pre-planned**	-9.39 (-10.37, -8.41)	-11.85 (-16.56, -7.13)	-7.15 (-8.07, -6.24)	-19.92 (-25.13, -14.70)	.225	.064	**.0046**	.255	.255	**<.0001**
	**Late-Cued**	-11.72 (-16.19, -7.26)	-6.97 (-18.06, 4.13)	-5.82 (-7.60, -4.05)	-41.05 (-51.08, -31.02)	.639	.075	**<.0001**	.826	**.003**	**<.0001**
**Participant 8**	**Straight**	-2.44 (-2.76, -2.11)	28.19 (26.14, 30.24)	1.49 (1.16, 1.83)	-25.98 (-27.81, -24.16)	<.0001	<.0001	**<.0001**	<.0001	**<.0001**	**<.0001**
	**Pre-planned**	-23.55 (-28.22, -18.88)	-20.53 (-27.89, -13.18)	-17.00 (-19.75, -14.24)	-66.33 (-76.84, -55.82)	.338	.222	**<.0001**	.449	**<.0001**	**<.0001**
	**Late-Cued**	-27.95 (-31.57, -24.32)	-14.91 (-22.64, -7.17)	-10.98 (-15.24, -6.73)	-103.17 (-110.59, -95.75)	**.011**	<.0001	**<.0001**	.494	**<.0001**	**<.0001**
**Participant 9**	**Straight**	-0.26 (-0.56, 0.04)	17.07 (15.26, 18.88)	0.34 (-0.63, 1.32)	-16.14 (-18.46, -13.82)	<.0001	.224	**<.0001**	<.0001	**<.0001**	**<.0001**
	**Pre-planned**	-13.19 (-15.03, -11.35)	-19.62 (-28.85, -10.40)	-14.82 (-17.68, -11.97)	-41.80 (-52.62, -30.99)	.283	.730	**.0002**	.730	.099	**.0001**
	**Late-Cued**	-18.15 (-22.22, -14.08)	-15.52 (-24.27, -6.77)	-11.85 (-15.80, -7.89)	-68.04 (-73.71, -62.38)	.955	.261	**<.0001**	.955	**<.0001**	**<.0001**
**Participant 10**	**Straight**	0.93 (0.76, 1.10)	12.54 (11.64, 13.45)	-1.44 (-1.63, -1.25)	-11.47 (-17.77, -10.41)	<.0001	<.0001	**<.0001**	<.0001	**<.0001**	**<.0001**
	**Pre-planned**	-7.98 (-9.27, -6.69)	-8.29 (-11.43, -5.16)	-7.20 (-8.49, -5.91)	-20.54 (-22.97, -18.12)	.999	.999	**<.0001**	.999	**<.0001**	**<.0001**
	**Late-Cued**	-14.09 (-17.77, -10.41)	-5.68 (-8.55, -2.82)	-4.07 (-5.11, -3.03)	-35.37 (-42.19, -28.55)	<.0001	<.0001	**<.0001**	.353	**<.0001**	**<.0001**

***Table SD2 4** Within-participant analysis for $F_{x,avg}$ (N) across gait phases within each task, where leftward $F_{x,avg}$ is negative and in the direction of the turn. Acronyms: Left Double Support (LDS) Left Single Support (LSS), Right Double Support (RDS), Right Single Support (RSS). Black text indicates hypothesis-specific gait phase comparisons. Bolded p-values indicate significant differences.*

		**Estimated Marginal Mean (95% CI)**				**Post-hoc comparisons**					
	**Task**	**LDS**	**LSS**	**RDS**	**RSS**	**LDS v. LSS**	**LDS v. RDS**	**LDS v. RSS**	**LSS v. RDS**	**LSS v. RSS**	**RDS v. RSS**
**Participant 1**	**Straight**	-4.50 (-7.46, -1.54)	29.07 (27.64, 30.49)	9.58 (7.29, 11.88)	-31.94 (-34.43, -29.45)	<.0001	<.0001	**<.0001**	<.0001	**<.0001**	**<.0001**
	**Pre-planned**	-72.15 (-78.51, -65.80)	-23.64 (-31.31, -15.96)	-62.04 (-71.37, -52.72)	-74.07 (-82.98, -65.16)	<.0001	.370	.790	<.0001	**<.0001**	**.0005**
	**Late - Cued**	-63.05 (-89.94, -36.17)	-15.46 (-38.26, 7.35)	-30.22 (-40.36, -20.09)	-107.92 (-127.77, -88)	<.0001	.117	.117	.290	**.0002**	**<.0001**
**Participant 2**	**Straight**	-3.32 (-4.95, -1.68)	15.35 (13.83, 16.87)	2.03 (0.22, 3.85)	-14.76 (-16.98, -12.54)	<.0001	**.003**	**<.0001**	<.0001	**<.0001**	**<.0001**
	**Pre-planned**	-46.08 (-52.40, -39.77)	-25.80 (-28.92, -22.68)	-41.13 (-45.47, -36.79)	-36.93 (-43.32, -30.54)	<.0001	.422	.177	**.0001**	**.019**	.422
	**Late - Cued**	-61.28 (-74.36, -48.19)	-37.70 (-59.98, -15.41)	-53.23 (-71.40, -35.06)	-69.25 (-98.00, -40.50)	.718	.999	.999	.436	.796	.999
**Participant 3**	**Straight**	-10.94 (-11.71, -10.17)	36.20 (33.53, 38.88)	8.97 (7.17, 10.77)	-34.06 (-37.77, -30.35)	<.0001	<.0001	**<.0001**	<.0001	**<.0001**	**<.0001**
	**Pre-planned**	-76.30 (-86.57, -66.03)	-33.02 (-41.00, -25.04)	-58.59 (-68.56, -48.62)	-81.84 (-97.50, -66.17)	<.0001	.146	.497	**.019**	**<.0001**	.090
	**Late-Cued**	-130.16 (-139.74, -120.57)	-38.13 (-47.74, -28.52)	-30.52 (-47.05, -13.99)	-134.79 (-86.57, -66.03)	<.0001	<.0001	.999	.999	**<.0001**	**<.0001**
**Participant 4**	**Straight**	-7.80 (-10.59, -5.02)	37.92 (36.05, 39.78)	-1.82 (-2.99, -0.66)	-34.72 (-35.68, -33.75)	<.0001	**.0017**	**<.0001**	<.0001	**<.0001**	**<.0001**
	**Pre-planned**	-42.57 (-47.19, -37.95)	-6.41 (-12.50, -0.32)	-39.77 (-44.95, -34.60)	-48.94 (-52.47, -45.41)	<.0001	.427	**.019**	<.0001	**<.0001**	**.006**
	**Late - Cued**	-45.63 (-57.16, -34.09)	-25.65 (-46.71, -4.59)	-42.87 (-56.13, -29.61)	-80.14 (-104.22, -56.06)	.317	.790	.083	.317	.086	**.086**
**Participant 5**	**Straight**	7.19 (6.49, 7.89)	20.34 (19.32, 21.37)	-6.04 (-6.59, -5.49)	-18.78 (-21.35, -16.22)	<.0001	<.0001	**<.0001**	<.0001	**<.0001**	**<.0001**
	**Pre-planned**	-26.49 (-32.67, -20.31)	-25.67 (-40.93, -10.42)	-41.86 (-50.34, -33.37)	-60.54 (-67.97, -53.12)	.875	.053	**<.0001**	.243	**.005**	**.0005**
	**Late-Cued**	-19.27 (-25.72, -12.82)	-10.50 (-25.10, 4.10)	-36.98 (-42.53, -31.43)	-72.76 (-86.86, -58.67)	.103	**.003**	**<.0001**	**.007**	**.0003**	**<.0001**
**Participant 6**	**Straight**	-7.08 (-8.54, -5.62)	31.26 (29.60, 32.92)	8.76 (7.39, 10.13)	-31.72 (-34.17, -29.27)	<.0001	<.0001	**<.0001**	<.0001	**<.0001**	**<.0001**
	**Pre-planned**	-77.66 (-88.20, -67.12)	-40.48 (-52.08, -28.89)	-54.65 (-61.88, -47.42)	-64.52 (-72.42, -56.62)	<.0001	**.034**	.186	.186	.054	.161
	**Late Cued**	-93.40 (-107.84, -78.97)	-52.90 (-79.35, -26.45)	-134.06 (-140.44, -127.68)	-77.66 (-88.20 (-67.12)	**.024**	**.0048**	**.0001**	.939	**<.0001**	**<.0001**
**Participant 7**	**Straight**	-6.41 (-7.80, -5.03)	32.80 (31.02, 34.58)	4.92 (3.33, 6.52)	-31.16 (-32.86, -29.46)	<.0001	<.0001	**<.0001**	<.0001	**<.0001**	**<.0001**
	**Pre-planned**	-52.01 (-57.37, -46.66)	-28.54 (-40.04, -17.04)	-39.68 (-44.51, -34.86)	-51.29 (-64.97, -37.62)	<.0001	.071	.935	.273	.273	.273
	**Late-Cued**	-54.87 (-73.04, -36.69)	-10.90 (-39.88, 18.09)	-34.07 (-44.40, -23.75)	-103.95 (-126.85, -81.04)	**.0002**	.114	**.003**	.114	**.002**	**.0002**
**Participant 8**	**Straight**	-13.66 (-15.13, -12.20)	65.28 (60.91, 69.64)	9.14 (7.51, 10.76)	-60.29 (-64.60, -55.97)	<.0001	<.0001	**<.0001**	<.0001	**<.0001**	**<.0001**
	**Pre-planned**	-117.70 (-139.54, -95.85)	-45.64 (-62.22, -29.07)	-94.71 (-113.55, -75.86)	-153.12 (-177.44, -128.80)	<.0001	.240	**.013**	**.013**	**<.0001**	**.013**
	**Late-Cued**	-124.94 (-143.24, -106.64)	-29.25 (-48.14, -10.36)	-72.37 (-103.16, -41.59)	-220.54 (-237.88, -203.20)	<.0001	**.001**	**<.0001**	.075	**<.0001**	**<.0001**
**Participant 9**	**Straight**	-3.40 (-5.05, -1.76)	42.42 (38.17, 46.66)	3.27 (-2.29, 8.83)	-40.23 (-46.13, -34.33)	<.0001	**.022**	**<.0001**	<.0001	**<.0001**	**<.0001**
	**Pre-planned**	-76.17 (-86.44, -65.90)	-45.32 (-66.65, -23.99)	-81.70 (-99.27, -64.13)	-100.65 (-126.17, -75.13)	**.0004**	.635	.372	.100	.081	.466
	**Late-Cued**	-92.02 (-109.22, -74.83)	-31.61 (-53.10, -10.11)	-64.51 (-88.45, -40.57)	-153.88 (-170.05, -137.71)	<.0001	.287	**.0005**	.287	**<.0001**	**<.0001**
**Participant 10**	**Straight**	3.95 (3.00, 4.90)	30.30 (28.12, 32.48)	-6.84 (-7.84, -5.84)	-27.57 (-29.40, -25.73)	<.0001	<.0001	**<.0001**	<.0001	**<.0001**	**<.0001**
	**Pre-planned**	-38.50 (-44.55, -32.45)	-17.16 (-24.17, -10.14)	-33.81 (-39.12, -28.51)	-44.83 (-50.88, -38.79)	<.0001	.284	.284	**.001**	**<.0001**	**<.0001**
	**Late-Cued**	-66.18 (-80.69, -51.66)	-9.93 (-17.13, -2.72)	-25.82 (-33.19, -18.44)	-66.19 (-85.24, -47.14)	<.0001	**.0009**	.999	**.048**	**.0002**	**<.00001**

Transverse-plane angular momentum (ΔHz) and average moment (Mz)

During straight-line gait, all 10 participants exhibited the group trends that ΔH_z and average M_z were greater during left double support vs. any other phase (p-values ≤ .0140). During pre-planned and during late-cued turns, all participants exhibited the group trend that the average M_z was greater during left double support vs. any other phase (p-values ≤.0001)

The group trend that the ΔH_z was greater during left double support vs. any other phase was exhibited by nine participants during pre-planned turns (p-values ≤ .0001) and seven participants during late-cued turns (p-values ≤ .0120).

Table SD2 5 *Within-participant analysis for ΔH_z (Nms) across gait phases within each task, where positive Hz indicates angular momentum as the body rotates leftward about a vertical axis through its center of mass. Acronyms: Left Double Support (LDS) Left Single Support (LSS), Right Double Support (RDS), Right Single Support (RSS). Black text indicates hypothesis-specific gait phase comparisons. Bolded p-values indicate significant differences.*

		Estimated Marginal Mean (95% CI)				Post-hoc comparisons					
	**Task**	**LDS**	**LSS**	**RDS**	**RSS**	**LDS v. LSS**	**LDS v. RDS**	**LDS v. RSS**	**LSS v. RDS**	**LSS v. RSS**	**RDS v. RSS**
	**Straight**	2.20 (2.10, 2.29)	-1.44 (-1.46, -1.41)	-2.50 (-2.60, -2.40)	1.74 (1.68, 1.79)	**<.0001**	**<.0001**	**<.0001**	<.0001	<.0001	<.0001
**Participant 1**	**Pre-planned**	2.40 (2.29, 2.50)	-1.59 (-1.69, -1.49)	-2.18 (-2.29, -2.06)	1.49 (1.41, 1.56)	**<.0001**	**<.0001**	**<.0001**	<.0001	<.0001	<.0001
	**Late - Cued**	1.85 (1.56, 2.14)	-1.56 (-1.72, -1.40)	-0.54 (-0.87, -0.21)	0.72 (0.51, 0.93)	**<.0001**	**<.0001**	**<.0001**	<.0001	<.0001	<.0001
	**Straight**	1.69 (1.63, 1.75)	-1.28 (-1.35, -1.20)	-1.98 (-2.02, -1.94)	1.59 (1.53, 1.64)	**<.0001**	**<.0001**	**.014**	<.0001	<.0001	<.0001
**Participant 2**	**Pre-planned**	1.70 (1.65, 1.75)	-1.34 (-1.39, -1.29)	-1.72 (-1.78, -1.66)	1.30 (1.21, 1.38)	**<.0001**	**<.0001**	**<.0001**	<.0001	<.0001	<.0001
	**Late - Cued**	1.70 (1.55, 1.84)	-1.40 (-1.81, -0.99)	-1.09 (-1.38, -0.80)	1.41 (1.12, 1.72)	**<.0001**	**<.0001**	.370	.370	<.0001	<.0001
	**Straight**	1.93 (1.81, 2.04)	-1.05 (-1.13, -0.97)	-1.48 (-1.57, -1.38)	0.58 (0.55, 0.61)	**<.0001**	**<.0001**	**<.0001**	<.0001	<.0001	<.0001
**Participant 3**	**Pre-planned**	2.33 (2.26, 2.40)	-0.96 (-1.05, -0.86)	-1.85 (-1.99, -1.72)	0.69 (0.53, 0.85)	**<.0001**	**<.0001**	**<.0001**	<.0001	<.0001	<.0001
	**Late-Cued**	2.62 (2.30, 2.94)	-1.71 (-1.92, -1.51)	-1.06 (-1.29, -0.83)	0.71 (0.55, 0.87)	**<.0001**	**<.0001**	**<.0001**	<.0001	<.0001	<.0001
	**Straight**	2.14 (2.03, 2.24)	-1.48 (-1.60, -1.35)	-2.30 (-2.33, -2.28)	1.65 (1.56, 1.75)	**<.0001**	**<.0001**	**<.0001**	<.0001	<.0001	<.0001
**Participant 4**	**Pre-planned**	1.93 (1.88, 1.99)	-1.33 (-1.41, -1.25)	-2.27 (-2.41, -2.14)	1.54 (1.44, 1.64)	**<.0001**	**<.0001**	**<.0001**	<.0001	<.0001	<.0001
	**Late - Cued**	1.89 (1.65, 2.14)	-1.47 (-1.69, -1.25)	-1.73 (-2.20, -1.26)	1.46 (1.07, 1.86)	**<.0001**	**<.0001**	.102	.285	<.0001	<.0001
	**Straight**	1.03 (0.98, 1.08)	-1.11 (-1.8, -1.03)	-1.44 (-1.50, -1.38)	1.53 (1.46, 1.59)	**<.0001**	**<.0001**	**<.0001**	<.0001	<.0001	<.0001
**Participant 5**	**Pre-planned**	1.22 (1.04, 1.39)	-1.17 (-1.38, -0.97)	-1.20 (-1.31, -1.10)	1.28 (1.01, 1.55)	**<.0001**	**<.0001**	.999	.999	<.0001	<.0001
	**Late-Cued**	1.12 (1.00, 1.24)	-1.10 (-1.23, -0.96)	-0.92 (-1.15, -0.68)	1.01 (0.86, 1.15)	**<.0001**	**<.0001**	.420	.420	<.0001	<.0001
	**Straight**	2.33 (2.26, 2.40)	-1.45 (-1.63, -1.28)	-2.76 (-2.85, -2.67)	1.87 (1.74, 2.00)	**<.0001**	**<.0001**	**<.0001**	<.0001	<.0001	<.0001
**Participant 6**	**Pre-planned**	2.63 (2.49, 2.77)	-1.73 (-1.87, -1.59)	-2.78 (-2.90, -2.66)	1.90 (1.79, 2.01)	**<.0001**	**<.0001**	**<.0001**	<.0001	<.0001	<.0001
	**Late Cued**	2.25 (2.06, 2.43)	-2.27 (-2.50, -2.04)	-1.15 (-1.41, -0.89)	1.50 (1.03, 1.97)	**<.0001**	**<.0001**	**.012**	<.0001	<.0001	<.0001
	**Straight**	1.94 (1.88, 2.00)	-0.49 (-0.59, -0.40)	-2.18 (-2.27, -2.10)	0.72 (0.60, 0.84)	**<.0001**	**<.0001**	**<.0001**	<.0001	<.0001	<.0001
**Participant 7**	**Pre-planned**	2.20 (2.08, 2.32)	-0.25 (-0.41, -0.10)	-2.42 (-2.57, -2.28)	0.66 (0.52, 0.80)	**<.0001**	**<.0001**	**<.0001**	<.0001	<.0001	<.0001
	**Late-Cued**	2.22 (2.00, 2.45)	-0.76 (-0.96, -0.56)	-1.25 (-1.68, -0.82)	0.26 (-0.01, 0.53)	**<.0001**	**<.0001**	**<.0001**	.063	<.0001	<.0001
	**Straight**	4.41 (4.31, 4.51)	-1.08 (-1.21, -0.95)	-4.37 (-4.53, -4.21)	1.05 (0.97, 1.13)	**<.0001**	**<.0001**	**<.0001**	<.0001	<.0001	<.0001
**Participant 8**	**Pre-planned**	5.52 (5.10, 5.93)	-1.74 (-2.04, -1.44)	-3.81 (-4.16, -3.46)	1.21 (0.95, 1.46)	**<.0001**	**<.0001**	**<.0001**	<.0001	<.0001	<.0001
	**Late-Cued**	4.48 (4.14, 4.82)	-2.53 (-2.86, -2.21)	-1.88 (-2.23, -1.53)	1.56 (1.14, 1.98)	**<.0001**	**<.0001**	**<.0001**	.014	<.0001	<.0001
	**Straight**	3.11 (2.89, 3.32)	-2.18 (-2.37, -1.99)	-3.61 (-3.80, -3.43)	2.67 (2.50, 2.84)	**<.0001**	**<.0001**	**.006**	<.0001	<.0001	<.0001
**Participant 9**	**Pre-planned**	3.30 (3.10, 3.51)	-2.21 (-2.46, -1.96)	-3.19 (-3.53, -2.85)	2.16 (1.77, 2.54)	**<.0001**	**<.0001**	**<.0001**	<.0001	<.0001	<.0001
	**Late-Cued**	3.08 (2.87, 3.29)	-2.44 (-2.75, -2.13)	-1.47 (-1.95, -0.98)	1.04 (0.21, 1.88)	**<.0001**	**<.0001**	**<.0001**	<.0001	<.0001	.0004
	**Straight**	2.28 (2.20, 2.36)	-1.23 (-1.31, -1.14)	-2.38 (-2.41, -2.34)	1.34 (1.27, 1.40)	**<.0001**	**<.0001**	**<.0001**	<.0001	<.0001	<.0001
**Participant 10**	**Pre-planned**	2.18 (2.11, 2.25)	-1.11 (-1.17, -1.05)	-2.24 (-2.28, -2.19)	1.27 (1.20, 1.34)	**<.0001**	**<.0001**	**<.0001**	<.0001	<.0001	<.0001
	**Late-Cued**	1.64 (1.53, 1.76)	-1.29 (-1.35, -1.24)	-1.58 (-1.82, -1.35)	1.23 (1.08, 1.37)	**<.0001**	**<.0001**	**.002**	.007	<.0001	<.0001

Table SD2 6 *Within-participant analysis for $M_{z,avg}$ (Nm) across gait phases within each task, where positive Mz indicates a moment that would tend to rotate the body leftward about a vertical axis through its center of mass. Acronyms: Left Double Support (LDS) Left Single Support (LSS), Right Double Support (RDS), Right Single Support (RSS). Black text indicates hypothesis-specific gait phase comparisons. Bolded p-values indicate significant differences.*

		**Estimated Marginal Mean (95% CI)**				**Post-hoc comparisons**					
	**Task**	**LDS**	**LSS**	**RDS**	**RSS**	**LDS v. LSS**	**LDS v. RDS**	**LDS v. RSS**	**LSS v. RDS**	**LSS v. RSS**	**RDS v. RSS**
**Participant 1**	**Straight**	14.49 (13.97, 15.01)	-3.71 (-3.77, -3.66)	-15.78 (-16.35, -15.22)	4.44 (4.29, 4.59)	**<.0001**	**<.0001**	**<.0001**	<.0001	<.0001	<.0001
	**Pre-planned**	15.12 (14.54, 15.71)	-3.91 (-4.18, -3.65)	-13.16 (-13.90, -12.42)	3.70 (3.48, 3.92)	**<.0001**	**<.0001**	**<.0001**	<.0001	<.0001	<.0001
	**Late - Cued**	9.62 (8.81, 10.43)	-4.24 (-4.82, -3.67)	-3.21 (-4.63, -1.78)	1.54 (1.13, 1.95)	**<.0001**	**<.0001**	**<.0001**	.253	<.0001	<.0001
**Participant 2**	**Straight**	10.63 (10.09, 11.17)	-3.40 (-3.63, -3.17)	-12.55 (-12.99, -12.12)	4.10 (3.93, 4.27)	**<.0001**	**<.0001**	**<.0001**	<.0001	<.0001	<.0001
	**Pre-planned**	9.44 (8.90, 9.99)	-3.44 (-3.58, -3.29)	-10.13 (-10.71, -9.54)	3.18 (2.93, 3.43)	**<.0001**	**<.0001**	**<.0001**	<.0001	<.0001	<.0001
	**Late - Cued**	7.26 (6.38, 8.13)	-3.33 (-4.45, -2.21)	-5.61 (-8.17, -3.04)	2.81 (2.17, 3.46)	**<.0001**	**<.0001**	**<.0001**	.194	<.0001	<.0001
**Participant 3**	**Straight**	10.18 (9.49, 10.87)	-2.59 (-2.79, -2.38)	-7.83 (-8.24, -7.42)	1.28 (1.20, 1.36)	**<.0001**	**<.0001**	**<.0001**	<.0001	<.0001	<.0001
	**Pre-planned**	12.75 (12.10, 13.40)	-2.28 (-2.54, -2.02)	-10.56 (-11.29, -9.83)	1.53 (1.12, 1.93)	**<.0001**	**<.0001**	**<.0001**	<.0001	<.0001	<.0001
	**Late-Cued**	11.61 (10.31, 12.90)	-4.09 (-4.65, -3.53)	-4.82 (-5.84, -3.80)	1.40 (1.07, 1.72)	**<.0001**	**<.0001**	**<.0001**	.194	<.0001	<.0001
**Participant 4**	**Straight**	11.03 (10.21, 11.85)	-3.78 (-4.09, -3.46)	-12.24 (-12.62, -11.86)	4.27 (4.02, 4.52)	**<.0001**	**<.0001**	**<.0001**	<.0001	<.0001	<.0001
	**Pre-planned**	9.60 (9.15, 10.05)	-3.18 (-3.34, -3.02)	-10.41 (-11.04, -9.78)	3.87 (3.61, 4.13)	**<.0001**	**<.0001**	**<.0001**	<.0001	<.0001	<.0001
	**Late - Cued**	9.35 (7.97, 10.73)	-3.40 (-3.86, -2.94)	-9.23 (-11.37, -7.08)	3.64 (2.70, 4.57)	**<.0001**	**<.0001**	**<.0001**	<.0001	<.0001	<.0001
**Participant 5**	**Straight**	5.73 (5.44, 6.03)	-2.75 (-2.94, -2.55)	-8.08 (-8.47, -7.68)	3.77 (3.58, 3.97)	**<.0001**	**<.0001**	**<.0001**	<.0001	<.0001	<.0001
	**Pre-planned**	7.28 (6.07, 8.48)	-2.88 (-3.39, -2.37)	-6.95 (-7.72, -6.19)	3.11 (2.41, 3.81)	**<.0001**	**<.0001**	**<.0001**	<.0001	<.0001	<.0001
	**Late-Cued**	6.01 (5.19, 6.83)	-2.77 (-3.15, -2.40)	-5.16 (-6.64, -3.68)	2.40 (2.05, 2.75)	**<.0001**	**<.0001**	**<.0001**	**.0048**	<.0001	<.0001
**Participant 6**	**Straight**	14.31 (13.93, 14.69)	-3.60 (-4.04, -3.16)	-16.97 (-17.87, -16.08)	4.61 (4.27, 4.96)	**<.0001**	**<.0001**	**<.0001**	<.0001	<.0001	<.0001
	**Pre-planned**	16.73 (15.46, 17.99)	-4.08 (-4.43, -3.73)	-17.95 (-19.16, -16.73)	4.70 (4.41, 4.99)	**<.0001**	**<.0001**	**<.0001**	<.0001	<.0001	<.0001
	**Late Cued**	13.43 (11.50, 15.36)	-4.99 (-5.74, -4.23)	-8.38 (-9.44, -7.32)	3.67 (2.48, 4.85)	**<.0001**	**<.0001**	**<.0001**	<.0001	<.0001	<.0001
**Participant 7**	**Straight**	10.37 (9.79, 10.95)	-1.15 (-1.40, -0.91)	-11.38 (-11.97, -10.80)	1.71 (1.40, 2.02)	**<.0001**	**<.0001**	**<.0001**	<.0001	<.0001	<.0001
	**Pre-planned**	12.04 (11.12, 12.96)	-0.47 (-0.86, -0.08)	-13.36 (-14.27, -12.45)	1.55 (1.17, 1.93)	**<.0001**	**<.0001**	**<.0001**	<.0001	<.0001	<.0001
	**Late-Cued**	11.45 (10.73, 12.18)	-1.89 (-2.43, -1.35)	-7.68 (-9.56, -5.80)	0.73 (-0.16, 1.63)	**<.0001**	**<.0001**	**<.0001**	<.0001	**.0002**	<.0001
**Participant 8**	**Straight**	21.18 (20.47, 21.90)	-2.35 (-2.65, -2.05)	-20.85 (-21.70, -20.00)	2.21 (2.04, 2.38)	**<.0001**	**<.0001**	**<.0001**	<.0001	<.0001	<.0001
	**Pre-planned**	27.03 (24.97, 29.08)	-3.63 (-4.26, -3.01)	-21.76 (-24.55, -18.97)	2.55 (1.95, 3.14)	**<.0001**	**<.0001**	**<.0001**	<.0001	<.0001	<.0001
	**Late-Cued**	20.56 (18.79, 22.33)	-5.58 (-6.39, -4.78)	-13.39 (-15.51, -11.27)	3.12 (2.36, 3.87)	**<.0001**	**<.0001**	**<.0001**	<.0001	<.0001	<.0001
**Participant 9**	**Straight**	18.00 (16.59, 19.4)	-5.23 (-5.75, -4.72)	-19.26 (-20.60, -17.92)	6.47 (6.02, 6.92)	**<.0001**	**<.0001**	**<.0001**	<.0001	<.0001	<.0001
	**Pre-planned**	19.18 (17.30, 21.07)	-4.99 (-5.53, -4.46)	-18.00 (-20.18, -15.81)	5.03 (3.99, 6.06)	**<.0001**	**<.0001**	**<.0001**	<.0001	<.0001	<.0001
	**Late-Cued**	16.15 (14.49, 17.82)	-5.64 (-6.31, -4.96)	-8.50 (-11.08, -5.93)	2.17 (0.20, 4.14)	**<.0001**	**<.0001**	**<.0001**	**.014**	<.0001	<.0001
**Participant 10**	**Straight**	12.63 (12.17, 13.09)	-2.83 (-3.05, -2.61)	-13.05 (-13.56, -12.54)	3.08 (2.94, 3.22)	**<.0001**	**<.0001**	**<.0001**	<.0001	<.0001	<.0001
	**Pre-planned**	10.40 (10.04, 10.76)	-2.36 (-2.51, -2.22)	-10.86 (-11.30, -10.43)	2.82 (2.61, 3.03)	**<.0001**	**<.0001**	**<.0001**	<.0001	<.0001	<.0001
	**Late-Cued**	7.74 (7.04, 8.44)	-2.81 (-2.96, -2.66)	-9.10 (-10.01, -8.20)	2.52 (2.30, 2.74)	**<.0001**	**<.0001**	**<.0001**	<.0001	<.0001	<.0001

SD 2.4 Preliminary exploration of controlling by the mean gait speed

When statistically controlling for average gait speed, the angular momenta group trends for ΔH_Z and $M_{Z,avg}$ and linear momenta group trends remain consistent with group trends that did not control for average gait speed. However, trends for $F_{x,avg}$ are different than those when the analysis did not control for average gait speed. At the moment, we believe that the observed $F_{x,avg}$ trial data overlap with other gait phases more than other study outcome variables (see main text **Figure 4 C & E**) Future studies can experimentally control for gait speed to better understand these phenomena. Until future studies can further investigate these relationships, readers are warned that statistically controlling for gait speed results in measures that were not actually observed. Humans may make different movement and momenta strategy choices at different gait speeds (which is not a research question this study was designed to answer).

***Table SD2** 7 Group-level estimated marginal means for study outcomes from linear mixed models controlling for average gait speed of the trial. Acronyms: Left Double Support (LDS) Left Single Support (LSS), Right Double Support (RDS), Right Single Support (RSS), change in transverse-plane angular momentum (ΔH_z), average transverse-plane moment ($M_{z,avg}$), where +Z is upward, change in linear momentum in the global X-direction (Δp_x), average global X-direction force ($F_{x,avg}$), where -X is leftward, in the direction of the turn. Black text indicates hypothesis-specific gait phase comparisons. Bolded p-values indicate significant differences.*

Outcome	Task	Estimated Marginal Mean (95% CI)				Post-hoc comparisons					
		**LDS**	**LSS**	**RDS**	**RSS**	**LDS v. LSS**	**LDS v. RDS**	**LDS v. RSS**	**LSS v. RDS**	**LSS v. RSS**	**RDS v. RSS**
ΔH_z	**Straight**	1.86 (1.60, 21.2)	-1.21 (-1.41, -1.01)	-2.00 (-2.52, -1.48)	1.23 (0.60, 1.86)	**<.0001**	**<.0001**	**.050**	**.014**	**<.0001**	**<.0001**
	**Pre-planned**	2.04 (1.56, 2.52)	-1.50 (-1.63, -1.38)	-1.91 (-2.20, -1.62)	1.38 (1.16, 1.61)	**<.0001**	**<.0001**	**.034**	**.034**	**<.0001**	**<.0001**
	**Late Cued**	2.09 (1.61, 2.57)	-1.57 (-1.71, -1.43)	-0.78 (-1.03, -0.54)	1.12 (0.65, 1.58)	**<.0001**	**<.0001**	**.009**	**<.0001**	**<.0001**	**<.0001**
Between Task p-values	**Straight v. Late-Cued**	.999	**.035**	**.0007**	.999						
	**Straight v. Pre-Planned**	.999	.064	.742	.999						
	**Late-Cued v. Pre-Planned**	.999	.431	**<.0001**	.857						
$M_{z,avg}$	**Straight**	11.62 (9.27, 13.97)	-3.17 (-3.88, -2.46)	-12.08 (-16.03, -8.12)	3.16 (1.34, 4.98)	**<.0001**	**<.0001**	**<.0001**	**.0002**	**<.0001**	**<.0001**
	**Pre-planned**	12.28 (8.11, 16.44)	-3.75 (-4.12, -3.38)	-11.60 (-13.76, -9.43)	3.42 (2.93, 3.93)	**<.0001**	**<.0001**	**.0003**	**<.0001**	**<.0001**	**<.0001**
	**Late Cued**	9.51 (7.30, 11.73)	-3.91 (-4.42, -3.40)	-4.59 (-6.31, -2.87)	2.25 (1.35, 3.14)	**<.0001**	**<.0001**	**<.0001**	.437	**<.0001**	**<.0001**
Between Task p-values	**Straight v. Late-Cued**	.590	.405	**.004**	.748						
	**Straight v. Pre-Planned**	.778	.450	.822	.765						
	**Late-Cued v. Pre-Planned**	.590	.519	**.0001**	.082						
ΔPx	**Straight**	-3.94 (-7.72, -0.17)	12.19 (8.64, 15.75)	0.12 (-1.40, 1.64)	-13.16 (-17.62, -8.69)	**<.0001**	**.034**	**.002**	**<.0001**	**<.0001**	**<.0001**
	**Pre-planned**	-9.99 (-13.88, -6.10)	-10.84 (-15.56, -6.12)	-6.96 (-8.01, -5.92)	-22.18 (-28.62, -15.74)	.771	.364	**.015**	.364	**.028**	**.001**
	**Late Cued**	-16.98 (-26.15, -7.81)	-15.05 (-20.34, -9.75)	-8.05 (-10.04, -6.07)	-39.43 (-48.31, -30.54)	.705	.124	**.007**	.055	**.0005**	**<.0001**
Between Task p-values	**Straight v. Late-Cued**	**.042**	**<.0001**	**<.0001**	**<.0001**						
	**Straight v. Pre-Planned**	.052	**<.0001**	**<.0001**	**.021**						
	**Late-Cued v. Pre-Planned**	.160	.230	.388	**.009**						
$F_{x,avg}$	**Straight**	-15.73 (-28.43, -3.04)	33.29 (24.62, 41.96)	4.07 (0.82, 7.32)	-36.04 (-48.12, -23.95)	**<.0001**	**.006**	**.011**	**<.0001**	**<.0001**	**<.0001**
	**Pre-planned**	-59.81 (-84.70, -34.92)	-27.90 (-41.67, -14.13)	-46.51 (-55.01, -38.02)	-56.66 (-73.10, -40.21)	.136	.847	.847	.136	.059	.847
	**Late Cued**	-77.94 (-119.37, -36.51)	-32.44 (-45.55, -19.33)	-44.98 (-56.81, -33.14)	-81.39 (-110.71, -52.06)	.162	.378	.888	.378	**.028**	.136
Between Task p-values	**Straight v. Late-Cued**	**.016**	**<.0001**	**<.0001**	**.025**						
	**Straight v. Pre-Planned**	**.008**	**<.0001**	**<.0001**	**.074**						
	**Late-Cued v. Pre-Planned**	.444	.625	.834	.146						
